# Olfactomedin-4 deletion exacerbates DSS-induced colitis through a matrix metalloproteinase-9-dependent mechanism

**DOI:** 10.7150/ijbs.80441

**Published:** 2023-04-09

**Authors:** Xinyu Wang, Shenghui Chen, Jinghua Wang, Yishu Chen, Yanjun Guo, Qinqiu Wang, Zhening Liu, Hang Zeng, Chengfu Xu

**Affiliations:** Department of Gastroenterology, the First Affiliated Hospital, Zhejiang University School of Medicine, Hangzhou 310003, China

**Keywords:** Ulcerative colitis, olfactomedin-4, matrix metalloproteinase-9, apoptosis

## Abstract

**Background and Aims:** Olfactomedin-4 is a glycoprotein that is upregulated in inflamed gastrointestinal tissues. This study aimed to investigate the role and underlying mechanisms of olfactomedin-4 in ulcerative colitis.

**Methods:** C57BL/6 mice and olfactomedin-4 knockout mice were fed dextran sulfate sodium in drinking water to establish a colitis model. An *in vitro* inflammation model was constructed in HCT116 and NCM460 cells stimulated with lipopolysaccharide. The expression of olfactomedin-4 was detected by Western blotting, immunohistochemistry staining, and qRT‒PCR. The differences in the severity of colitis between olfactomedin-4 knockout mice and wild-type mice were compared, and the underlying mechanisms were explored.

**Results:** Olfactomedin-4 expression was significantly upregulated in colonic tissues of active ulcerative colitis patients and in cellular and mouse models of colitis. Compared with wild-type littermates, olfactomedin-4 knockout mice were more susceptible to dextran sulfate sodium-induced colitis and produced higher levels of proinflammatory cytokines and chemokines. In addition, olfactomedin-4 deficiency significantly promoted intestinal epithelial cell apoptosis and increased intestinal permeability, which was mediated by the p53 pathway. Moreover, olfactomedin-4 directly interacted with and negatively regulated matrix metalloproteinase-9. Inhibiting matrix metalloproteinase-9 significantly decreased colonic p53 expression and ameliorated experimental colitis in olfactomedin-4 knockout mice, while overexpression of matrix metalloproteinase-9 aggravated colitis. Further experiments showed that matrix metalloproteinase-9 regulated p53 through the Notch1 signaling pathway to promote ulcerative colitis progression.

**Conclusions:** Olfactomedin-4 is significantly upregulated in ulcerative colitis and may protect against colitis by directly inhibiting matrix metalloproteinase-9 and further decreasing p53-mediated apoptosis *via* Notch1 signaling.

## Introduction

Ulcerative colitis (UC) is one of the two major types of inflammatory bowel disease (IBD), which is a chronic intestinal inflammatory disorder characterized by diarrhea, abdominal pain, and rectal bleeding 1. Accumulating evidence suggests that UC results from multiple causes, including genetics, the environment, microbes, and inappropriate inflammatory responses 1-3. Dysregulation of immune responses is considered to play a major role in UC development 4, 5. Various proinflammatory cytokines and chemokines play a central role in abnormal immune responses 5, 6. Moreover, intestinal epithelial cells (IECs) provide the first-line defense formed by a physical and biochemical barrier 7. Excessive apoptosis of IECs 8, 9, breakage of tight junction complexes 10, defects in the mucus layer 11, and any other behaviors leading to a compromised barrier with bacterial invasion may create a vicious cycle exacerbating UC 11-13. However, the exact pathogenesis of UC is still under investigation.

Olfactomedin-4 (OLFM4, also called GW112 or hGC-1), a 72 kDa glycoprotein belonging to the olfactomedin family, is highly expressed in the small intestine and colon 14, 15. OLFM4 is widely distributed in cells, including the membrane, cytoplasm, nucleus, and mitochondria 16, 17. OLFM4 interacts with several other proteins to perform a variety of biological functions, including proliferation, differentiation, apoptosis, and cell adhesion 17, 18. This molecule also plays an important role in innate immune regulation 19. In addition, OLFM4 can be secreted into the extracellular space for biological purposes 20. The expression of OLFM4 is upregulated in the intestinal epithelium of IBD patients, including those with UC and Crohn's disease (CD), but is more significantly upregulated in active UC patients 21. Previous studies have shown that the OLFM4 protein marks intestinal stem cells 22 and is aberrantly expressed in the inflamed colonic mucosa in UC patients 21, 23. However, the specific functions of OLFM4 in the colon are still unclear.

Matrix metalloproteinase-9 (MMP9, gelatinases B) is a multidomain enzyme belonging to the MMP family 24. In addition to the conserved catalytic domain of MMPs, MMP9 contains a unique O-glycosylated domain that can facilitate enzyme flexibility 25. Strong evidence indicates that MMP9 induces chronic inflammation, aberrant tissue remodeling, and degradation of extracellular matrix (ECM) components 26, 27, which are hallmarks of IBD. The expression of MMP9 was found in the mucosal tissue of IBD patients, most prominently in actively inflamed areas 28, 29. The serum neutrophil gelatinase B-associated lipocalin and MMP9 (Ngal-MMP9) complex is suggested as a surrogate marker to assess mucosal healing in UC and Crohn's disease patients 30, 31. However, the upstream and downstream molecules involved in the mechanisms of MMP9 in UC remain unclear.

The multifunctional gene p53 acts as a tumor suppressor to determine cell fate and prevent the expansion of damaged cells 32. Increased expression of p53 has been detected in UC patients and animal colitis models 33, 34. Studies have shown that p53 is required for IEC apoptosis 35, which requires the expression of p53 and p53 upregulated modulator of apoptosis (PUMA) 8, 36. Several groups have revealed that p53 deficiency could protect against acute intestinal inflammation 33, 36. The regulatory effect of MMP9 on p53 has been reported and involves Notch signaling 37, 38. However, the involvement of MMP9- and p53-mediated apoptosis in ulcerative colitis has not been fully explored.

In this study, we aimed to explore the role and underlying regulatory mechanism of OLFM4 in ulcerative colitis.

## Materials and methods

### Human tissue

Human colonic mucosa specimens from 17 active UC patients and 8 healthy controls were obtained during endoscopic examinations at the Department of Gastroenterology, the First Affiliated Hospital, Zhejiang University School of Medicine. The diagnosis of UC was based on accepted clinical criteria, including typical symptoms, as well as endoscopic, histologic, and radiographic diagnoses 39. This study was approved by the Clinical Research Ethics Committee of the First Affiliated Hospital, Zhejiang University School of Medicine (approval no. 2022354).

### Animal treatment

OLFM4 knockout mice (*Olfm4*-/-) on a C57BL/6 background were generated through CRISPR technology by Beijing ViewSolid Biotechnology (Beijing, China). Wild-type littermate male C57BL/6 mice were purchased from Zhejiang Experimental Animal Centre (Hangzhou, China). Mice were acclimated for 7 days in an animal room with air-conditioned specific pathogen-free (SPF) conditions at 23± 2 °C with a 12 h light/dark cycle before experimentation. The protocol to develop chemically induced acute colitis in C57BL/6 mice or *Olfm4*-/- mice was described previously 40. The male mice (8 weeks old) received 35 g/L dextran sulfate sodium (DSS, 36-50 kDa, MP Biomedicals, Santa Ana, CA) in their drinking water for 6 days, while control mice received autoclaved water. The body weight, the presence of occult or gross blood per rectum, and stool consistency were assessed daily for the calculation of the disease activity index score, as previously described 41. Entire colons were collected from the experimental mice and fixed flat in 40 g/L formaldehyde for 48 h. Colon sections (5 mm thick) were embedded in paraffin and stained with hematoxylin and eosin. The inflammation scores were determined as previously described 41, 42. All animal studies were approved by the Animal Care and Use Committee of the First Affiliated Hospital, Zhejiang University School of Medicine (approval No. 20191097).

### Adeno-associated virus (AAV) administration

Recombinant AAV serotype 9 was selected, according to the literature, to mediate relatively high efficiency gene delivery to intestinal epithelial cells 43, 44. AAV carrying shMMP9 (AAV-shMMP9) or control vector (AAV-shNC) was generated and purified as described previously 45. Then, male 8-week-old wild-type (WT) and *Olfm4*-/- mice (n = 7-10/group) received a single intravenous dose of 2 × 1011 AAV-shMMP9 or AAV-shNC in a volume of 150 µl through tail vein injection according to previous studies 43, 46. All mice were housed under a standard 12-hour light/dark cycle and fed a standard rodent chow diet and autoclaved tap water ad libitum. After 1 week, colitis was induced by DSS in drinking water for 6 days.

### Inhibition of Notch signaling

Difluorophenacetyl-L-alanyl-S-phenylglycine t-butyl ester (DAPT, Sigma‒Aldrich) was used to inhibit the Notch signaling pathway 47. HCT116 cells were treated with 1 μmol/ml DAPT. Age- and sex-matched C57B6 WT and *Olfm4*-/- mice (n=10/group) were obtained by intraperitoneal injection of DAPT five consecutive times during DSS drinking to inhibit Notch signaling. The *in vivo* effects of Notch inhibitors in various mouse disease models have been well studied 38, 48, 49. We chose 10 μmol/kg DAPT as described in previous studies, while the controls received vehicle alone 38. DAPT was solubilized in DMSO and diluted in phosphate-buffered saline (PBS) containing 0.01% (v/v) Tween 80 and 0.5% (w/v) hypromellose (GlpBio, Montclair, CA) 38.

### Cell culture and *in vitro* model

The human colorectal adenocarcinoma cell line HCT116, human embryonic kidney 293T cells, and normal colonic epithelial cell line NCM460 (Institute of Biochemistry and Cell Biology, China Academy of Sciences, Shanghai, China) were used for experiments. HCT116 and 293T cells were cultivated in Dulbecco's modified Eagle's medium (DMEM, Gibco Life Technologies, Eggenstein, Germany), and NCM460 cells were cultured in Roswell Park Memorial Institute (RPMI)-1640 medium. The cells were cultured in medium with 100 ml/L fetal bovine serum (FBS, Invitrogen, Carlsbad, CA) and 10 ml/L penicillin‒streptomycin (Sigma‒Aldrich, St. Louis, MO) in a humidified atmosphere at 37 °C and 50 mL/L CO2. Cells were exposed to lipopolysaccharide (LPS, 1 μg/ml) (Sigma‒Aldrich, St. Louis, MO) to establish the *in vitro* inflammation model 50, 51.

### Gene expression measurements

mRNA was isolated from cells or tissues using RNAiso Plus (TaKaRa, Otsu, Japan). Prime Script RT Master Mix (TaKaRa) was used to generate cDNA from mRNA. Quantitative real-time PCR was performed using TB Green Premix Ex Taq II (TaKaRa) on a 7500 Fast Real-Time PCR system (Applied Biosystems, Foster City, CA). For the specific primer sequences, see Supplementary Table S1.

### Cell transfection

The siRNA oligonucleotides and overexpression plasmids of OLFM4 were purchased from RiboBio (Guangzhou, China). The siRNA, plasmids and corresponding negative controls (at a final concentration of 5 nM) were transfected into cells using Lipofectamine 3000 (Invitrogen) according to the manufacturer's protocol 52. Cells were transfected with siRNAs for 24 h or plasmid DNA for 6 h and then exposed to LPS for another 24 h.

### Western blot assays

Protein was isolated from cells and mouse distal colon tissue by RIPA lysis buffer (Applygen Technologies, Beijing, China) containing protease and phosphatase inhibitors (Pierce Biotechnology, Rockford, IL). Western blotting was carried out as previously described. The protein (20 μg/sample) was separated on an 80 g/L-100 g/L SDS‒PAGE gel and transferred to a polyvinylidene difluoride membrane (0.2 mm pore; Millipore, Darmstadt, Germany). Membranes were preincubated with 50 g/L nonfat powdered milk in TBST for 1.5 h, followed by incubation with specific primary antibodies at 4 °C overnight. The primary antibodies are listed in Supplementary Table S2. Signals were detected by HRP-conjugated secondary antibodies (Santa Cruz, CA), and densitometric analysis was performed using ImageJ software (Version 1.51).

### Enzyme-linked immunosorbent assay (ELISA)

The protein levels of cytokines and chemokines were tested by ELISAs as previously described 53. Serum IL-1β, IL-6, MCP1, and ICAM1 levels were measured using commercially available ELISA kits (Mouse IL1β ELISA Kit, KE10003, Proteintech; Mouse IL6 ELISA kit, KE10007, Proteintech; Mouse MCP1 ELISA Kit, KE10006, Proteintech; Mouse ICAM1 ELISA Kit, KE10063 Proteintech) following the manufacturer's instructions.

### Immunohistochemistry (IHC)

For IHC, after blocking with 100 g/L normal goat serum (ZSGB-BIO, Beijing, China) in PBS (pH 7.5), tissue sections were incubated with primary antibodies against OLFM4 (dilution: 1:200, Cell Signaling Technology, Danvers, MA) overnight at 4 °C. Tissue sections were stained with secondary antibodies (dilution: 1:1000, ZSGB-BIO, Beijing, China) for 1 h in an incubator maintained at 37 °C. Immunoreactivity was detected by using a DAB kit (ZSGB-BIO).

### Terminal deoxynucleotidyl transferase-mediated deoxyuridine triphosphate nick-end labeling (TUNEL) staining

For detection of apoptosis, sections of tissue were stained by TUNEL with a cell death detection kit (Roche, Indianapolis, IN) as described previously 54. Sections were counterstained with DAPI. Fluorescence images were captured by confocal microscopy (Olympus Corporation, Japan). The number of TUNEL-positive epithelial cells was quantified, and the percentage of apoptotic cells was calculated from eight enlarged photomicrographs of the colon as previously reported 33, 36.

### Fluorescein isothiocyanate (FITC)-dextran permeability assay

Intestinal permeability was assessed by oral administration of FITC-labeled dextran (MW 4,000; Sigma‒Aldrich) as previously described 55. After the withdrawal of food overnight, all mice were gavaged with FITC-dextran (60 mg/100 g of body weight) 4 h before sacrifice. The serum was then collected, and the FITC-dextran level in the serum was measured with a fluorescence spectrophotometer with emission and excitation wavelengths of 488 nm and 520 nm, respectively.

### RNA sequencing

Total RNA was extracted using TRIzol (TaKaRa) according to the manufacturer's protocol. Preparation of the library and transcriptomic sequencing were carried out using the Illumina HiSeq ×Ten (Novogene Bioinformatics Technology). Mapping of 100-bp paired-end reads to genes was performed using HTSeq software (version 0.6.0), and fragments per kilobase of transcript per million fragments mapped (FPKM) were also analyzed.

### Statistical analysis

Statistical analyses were performed, and all graphs were generated with GraphPad Prism software version 8.2.0 using one-way analysis of variance (ANOVA). All data are expressed as the mean ± standard deviation (SD) from at least three independent experiments. P values less than 0.05 were considered statistically significant.

### Ethics Approval

This study was approved by the Clinical Research Ethics Committee of the First Affiliated Hospital, Zhejiang University School of Medicine. The experimental procedures were performed in accordance with the approved guidelines of the Ethics Committee. All animal studies were approved by the Animal Care and Use Committee of the First Affiliated Hospital, Zhejiang University School of Medicine and were administered in accordance with the Chinese guidelines for the care and use of laboratory animals.

## Results

### OLFM4 is highly expressed in inflamed but not normal colon tissue and cells

**Figure 1:**
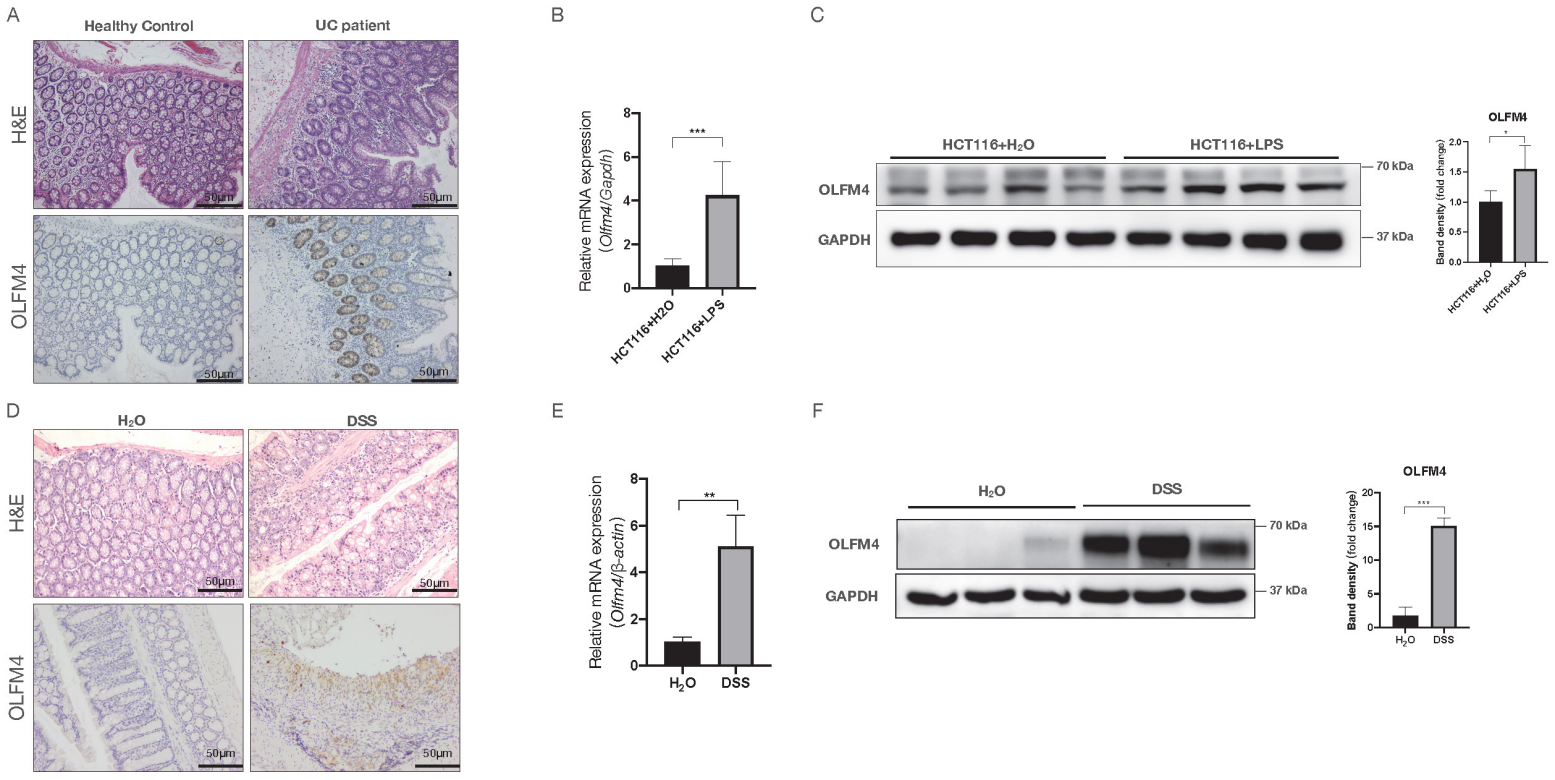
** OLFM4 expression is upregulated in colitis both* in vivo* and *in vitro.* (A)** H&E-stained images of colon tissues from UC patients and healthy controls (above). Immunohistochemistry of OLFM4 is described in the figure below (scale bar: 50 μm). **(B)** qRT‒PCR analysis of* Olfm4* mRNA levels in LPS-stimulated HCT116 cells. Data are the means ± SDs. *n* = 6. ***P<0.001. **(C)** Western blotting analysis of OLFM4 protein levels in LPS-stimulated HCT116 cells. The OLFM4 protein level was quantified using ImageJ software analysis. *P<0.05. **(D)** H&E-stained images of colon sections from WT mice (above). Immunohistochemistry of OLFM4 is described in the figure below (scale bar: 50 μm). **(E)** *Olfm4* mRNA levels were measured in distal colons by qRT‒PCR. Data are the means ± SDs. *n* = 5-8. **P<0.01. **(F)** The protein level of OLFM4 was measured in distal colons by Western blots and quantified by ImageJ software. ***P<0.001.

To investigate the potential association of OLFM4 with UC, we analyzed OLFM4 protein expression in colon tissues from active UC patients. Compared with the healthy controls, the UC patients showed significantly higher colonic expression of OLFM4, as indicated by immunohistochemistry staining (Figure 1A). We also found that OLFM4 expression was significantly increased at both the mRNA and protein levels in LPS-stimulated HCT116 and NCM460 cells (Figure 1B and 1C; Figure S1A and S1B). Moreover, we established a DSS-induced mouse model of acute colitis (Figure 1D; Figure S1C and S1D) and found that OLFM4 levels were significantly increased in the inflammatory area of the colon (Figure 1D). qRT‒PCR and Western blotting further confirmed that OLFM4 was highly expressed in the distal colon of the DSS-treated mice but barely expressed under normal conditions (Figure 1E and 1F). These findings suggested a potential association of OLFM4 with UC.

### *Olfm4* deficiency exacerbates inflammation both *in vitro* and* in vivo*

**Figure 2:**
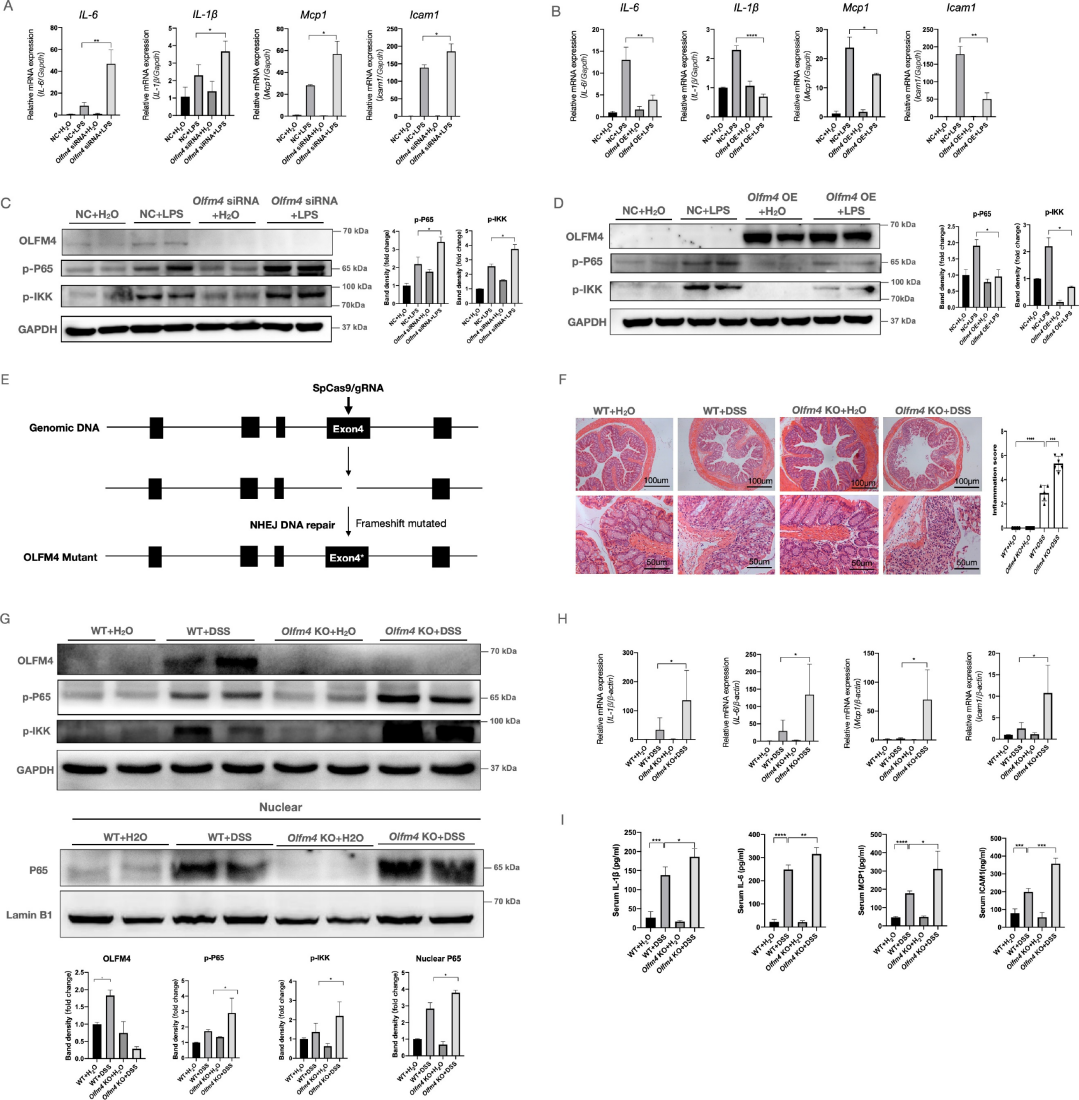
** Deletion of *Olfm4* exacerbates colitis. (A)** The mRNA levels of *Il6, Il1β, Mcp1*, and *Icam1* were measured in HCT116 cells by qRT‒PCR. Data are the means ± SDs. *n* = 6. *P<0.05, **P<0.01. **(B)** The mRNA levels of *Il6, Il1β, Mcp1*, and *Icam1* were measured in HCT116 cells by qRT‒PCR. Data are the means ± SDs. *n* = 6. *P<0.05, **P<0.01, ***P<0.001. **(C)** The protein levels of OLFM4, p-P65, and p-IKK were measured in HCT116 cells by Western blotting and quantified by ImageJ software. *P<0.05. **(D)** The protein levels of OLFM4, p-P65, and p-IKK were measured in HCT116 cells by Western blot and quantified by ImageJ software. *P<0.05. **(E)** *Olfm4^-/-^* mice were generated through CRISPR technology. **(F)** Colon sections from WT mice and *Olfm4* KO mice were stained with H&E (left), and inflammation scores (right) were determined to assess injury and inflammation. ***P<0.001, ****P<0.0001. **(G)** The protein levels of OLFM4, p-P65, and p-IKK and the nuclear level of P65 were measured in the distal colons of WT mice and *Olfm4^-/-^* mice by Western blotting and quantified by ImageJ software. *P<0.05. **(H)** The mRNA levels of *Il1β, Il6, Mcp1*, and *Icam1* were measured in distal colons by qRT‒PCR. Data are the means ± SDs. *n* = 5-8. *P<0.05. **(I)** The protein levels of IL-1β, IL-6, MCP1, and ICAM1 were measured in serum by ELISAs. Data are the means ± SDs. *n* = 5-8. *P<0.05, **P<0.01, ***P<0.001, ****P<0.0001.

To explore the function of OLFM4 in colitis, we assessed the effects of OLFM4 knockdown or overexpression on LPS-stimulated inflammation in HCT116 cells. As determined by qRT‒PCR, *Olfm4* expression in HCT116 and NCM460 cells was successfully inhibited by *Olfm4* siRNA and significantly increased by the overexpression plasmids (Figure S2A-S2D). Inhibition of *Olfm4* accelerated LPS-stimulated inflammation, while overexpression of *Olfm4* decreased the production of LPS-stimulated inflammatory cytokines and chemokines, including interleukin-6 (Il6), Il1β, monocyte chemoattractant protein-1 (Mcp1), and intercellular adhesion molecule-1 (Icam1) (Figure 2A and 2B; Figure S2E). Icam1 is a downstream factor of the nuclear factor (NF)-κB pathway 56, which plays an important role in colitis 57. We found that knocking down *Olfm4* significantly increased the phosphorylation of the NF-κB subunit p65 and the IκB kinase (IKK) complex in LPS-stimulated cells (Figure 2C; Figure S2F). In contrast, *Olfm4* overexpression significantly decreased LPS-induced NF-κB pathway activation (Figure 2D).

To further explore the *in vivo* function of *Olfm4* in colitis, we generated *Olfm4*-/- mice and established a mouse model of acute colitis by adding 35 g/L DSS to the drinking water (Figure 2E; Figure S3A). We found that body weight (Figure S3B), colon length (Figure S3C), and H&E staining (Figure 2F) all indicated more severe and extensive colitis in the *Olfm4*-/- mice than in the WT littermates. The phosphorylated levels of p65, nuclear transport of p65, and the IKK complex were also markedly increased in the colons of the DSS-treated *Olfm4*-/- mice compared with those in the WT controls (Figure 2G). The *Olfm4*-/- mice also had significantly higher colonic mRNA expression and serum levels of proinflammatory cytokines and chemokines, including IL-1β, IL-6, MCP1, and ICAM1, than the WT mice after 6 days of DSS challenge (Figure 2H and 2I). These results suggest that *Olfm4* deficiency exacerbates colitis both *in vitro* and *in vivo*.

### *Olfm4* deficiency promotes IEC apoptosis and increases intestinal permeability

**Figure 3:**
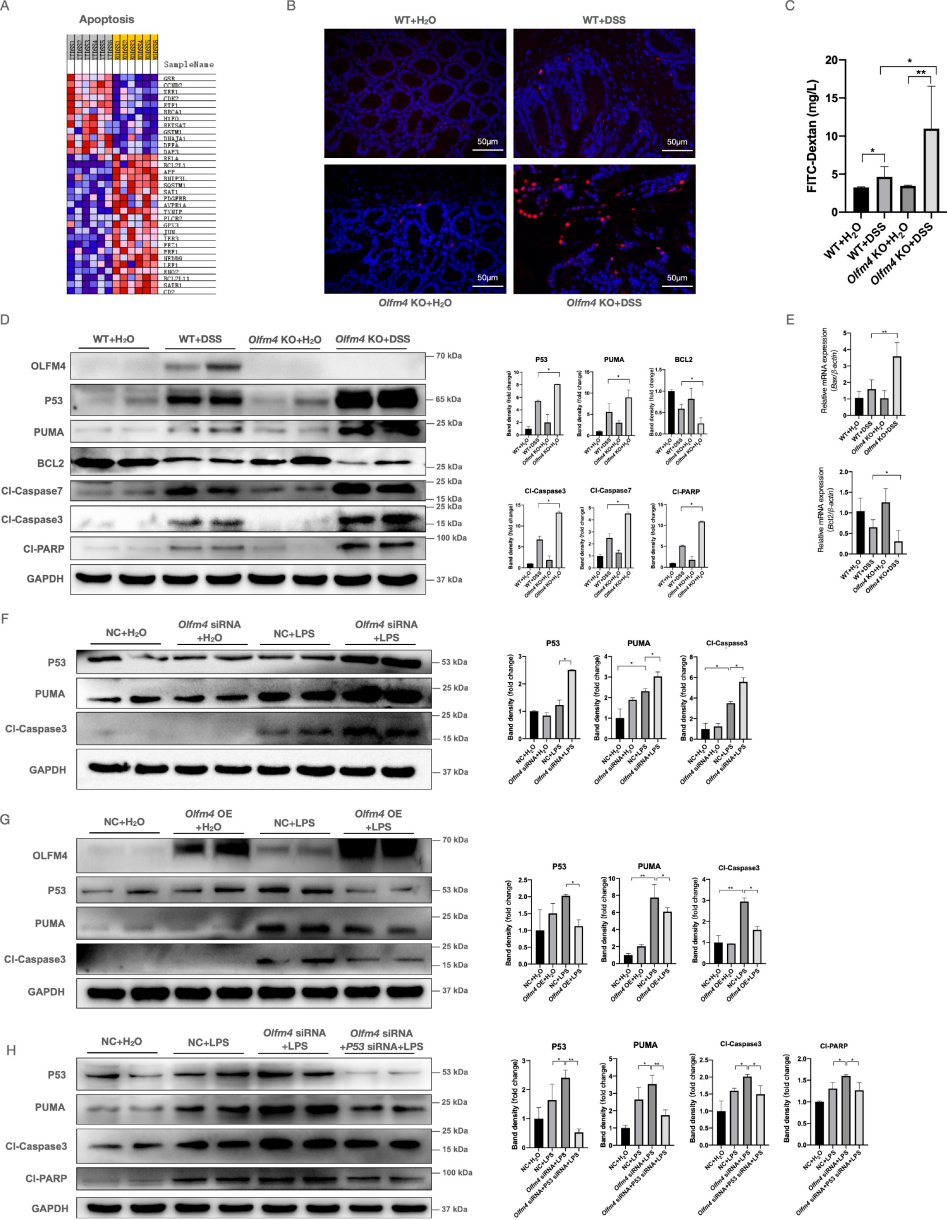
***Olfm4* deficiency promotes p53-mediated IEC apoptosis. (A)** RNA sequencing detected differentially expressed mRNAs in DSS-treated WT and *Olfm4*^-/-^ mice. **(B)** TUNEL-positive apoptotic cells in the colons of WT and *Olfm4^-/-^* mice upon DSS treatment. **(C)** Serum FITC-dextran levels of DSS-treated WT and *Olfm4^-/-^* mice. *n* = 5-8. *P<0.05, **P<0.01. **(D)** Representative WB analyses (left) and quantification (right) of the protein levels of genes related to p53-mediated apoptosis in mice. *P<0.05. **(E)** The mRNA levels of* Bax* and *Bcl2* were measured in distal colons by qRT‒PCR. Data are the means ± SDs. *n* = 5-8. *P<0.05, **P<0.01. **(F)** Representative western blot analyses (left) and quantification (right) of the protein levels of genes related to p53-mediated apoptosis in HCT116 cells. *P<0.05. **(G)** Representative western blot analyses (left) and quantification (right) of the protein levels of genes related to p53-mediated apoptosis in HCT116 cells. *P<0.05, **P<0.01. **(H)** Representative WB analyses (left) and quantification (right) of the protein levels of genes related to p53-mediated apoptosis in LPS-induced HCT116 cells simultaneously transfected with *Olfm4* siRNA and p53 siRNA. *P<0.05, **P<0.01.

We performed next-generation RNA sequencing to explore the regulatory mechanisms of OLFM4 in colitis. As expected, numerous differentially expressed transcripts were observed in the colon of the DSS-treated *Olfm4*-/- mice compared with the DSS-treated WT mice. Gene set enrichment analysis (GSEA) identified a specific role of *Olfm4* in the apoptosis pathway (Figure 3A). Because excessive IEC apoptosis is closely associated with increased intestinal barrier permeability and deteriorated UC symptoms 8, 9, we assessed whether *Olfm4* affects IEC apoptosis and regulates intestinal barrier integrity. We found that the *Olfm4*-/- mice showed more TUNEL-positive apoptotic cells in the colon than the WT mice upon DSS challenge (Figure 3B). We also observed a threefold increase in the serum FITC-dextran levels of the *Olfm4*-/- mice after 6 days of DSS challenge compared with the DSS-treated WT mice (Figure 3C). Moreover, the levels of cleaved caspase 3 and caspase 7, two activated forms of the most important downstream caspases in the apoptosis pathway, were significantly higher in the *Olfm4*-/- mice than in the WT mice after DSS challenge (Figure 3D). Other apoptosis-related factors, including cleaved poly (ADP-ribose) polymerase (PARP) and B-cell lymphoma 2 (BCL2)-associated X protein (BAX), were also increased, while the antiapoptotic factor BCL2 was significantly decreased in the DSS-treated *Olfm4*-/- mice compared with the WT controls (Figure 3D and 3E). Additionally, we examined other cell death mechanisms, including necroptosis and ferroptosis. *Olfm4* deficiency did not affect these processes (Figure S4A and S4B). These findings suggested that *Olfm4* deficiency promotes IEC apoptosis and increases intestinal permeability in the mice with DSS-induced colitis.

### *Olfm4* deficiency promotes IEC apoptosis by enhancing p53 activation

We further investigated the mechanism by which OLFM4 regulates IEC apoptosis. P53 is a key regulator of apoptosis, the cell cycle, and senescence 58, and it is involved in IBD pathogenesis by regulating cell cycle arrest and apoptosis 12, 36, 42. Here, we found that *Olfm4* deficiency significantly upregulated the colonic protein p53 and its downstream factor, PUMA, in DSS-treated mice (Figure 3D). We also found that knockdown of *Olfm4* significantly increased the expression of p53 and PUMA in LPS-stimulated HCT116 cells (Figure 3F), while overexpression of *Olfm4* inhibited p53 pathway activation (Figure 3G). Moreover, siRNA-mediated knockdown of p53 inhibited the excessive activation of the apoptosis pathway caused by *Olfm4* knockdown in LPS-stimulated HCT116 cells (Figure 3H). These results indicate that *Olfm4* deficiency may promote IEC apoptosis by enhancing p53 activation and may thereby exacerbate colitis.

### MMP9 mediates the regulatory effect of OLFM4 on p53 in colitis

**Figure 4:**
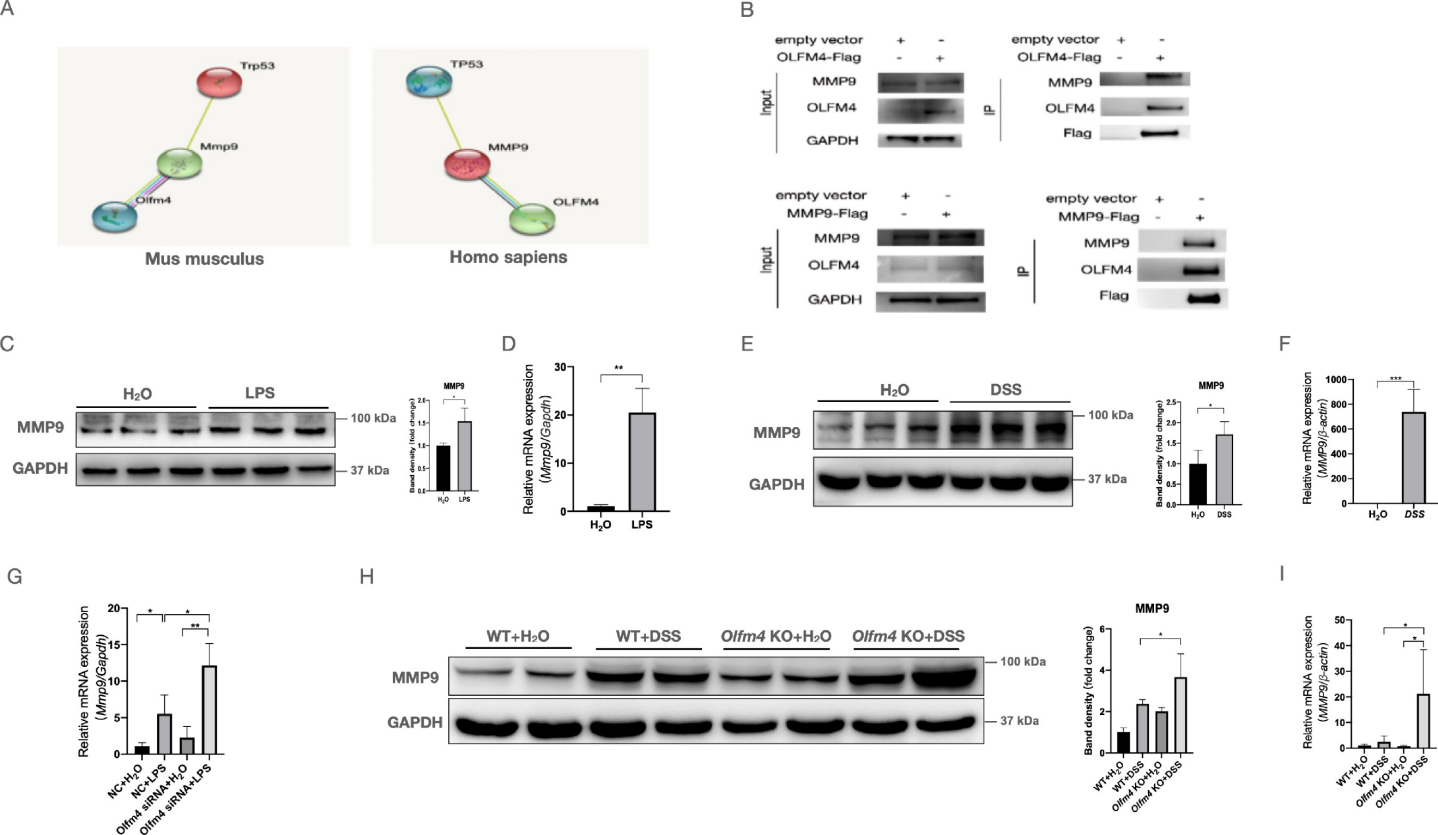
** MMP9 mediates the interaction between OLFM4 and p53. (A)** The relationship predicted by the STRING database (http://string-db.org/). **(B)** Co-IP analysis confirmed the interaction between OLFM4 and MMP9 in 293T cells. **(C)** The protein levels of MMP9 in HCT116 cells measured by Western blots. *P<0.05. **(D)** The mRNA expression of MMP9 in HCT116 cells. Data are the means ± SDs. *n* = 6. **P<0.01. **(E)** The protein levels of MMP9 in the colons of DSS-treated WT mice compared with control mice. *P<0.05. **(F)** The mRNA expression of *Mmp9* in mouse colons. Data are the means ± SDs. *n* = 6. ***P<0.001. **(G)** The mRNA expression of MMP9 was detected in HCT116 cells by qRT‒PCR. Data are the means ± SDs. *n* = 6. *P<0.05, **P<0.01. **(H)** The protein levels of MMP9 were detected in the colons of DSS-treated WT or *Olfm4^-/-^* mice by Western blotting analysis. *P<0.05. **(I)** The mRNA expression of *Mmp9* was detected in the colons of DSS-treated WT or *Olfm4^-/-^* mice by qRT‒PCR. Data are the means ± SDs. *n* = 6. *P<0.05.

To investigate whether OLFM4 regulates p53 directly or indirectly, we searched the STRING database (http://string-db.org/) and predicted MMP9 to mediate the interaction between OLFM4 and p53 (Figure 4A). Our coimmunoprecipitation (co-IP) analysis confirmed the interaction between OLFM4 and MMP9 in 293T cells and HCT116 cells (Figures 4B and S5A). We also observed that the expression of MMP9 was dramatically upregulated at both the mRNA and protein levels in the cellular and mouse models of colitis (Figure 4C-4F), and knockout of *Olfm4* further upregulated the expression of MMP9 in LPS-stimulated cells and in the colon of DSS-treated mice (Figure 4G-4I; Figure S5B).

**Figure 5:**
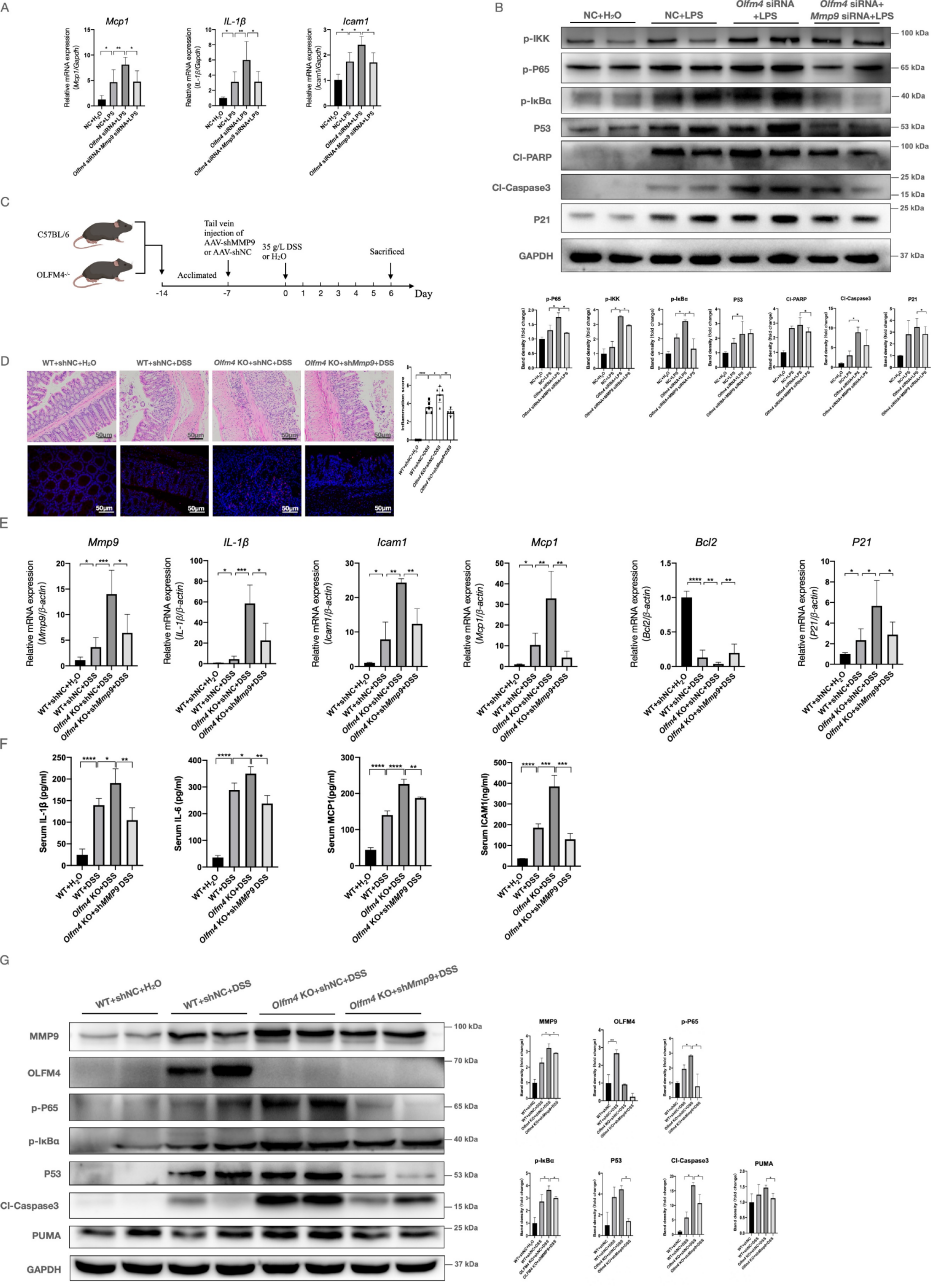
** MMP9 mediates the regulatory effect of OLFM4 on p53. (A)** The mRNA expression of *Mcp1*, *Il1β,* and *Icam1* was detected in HCT116 cells by qRT‒PCR. Data are the means ± SDs. *n* = 6. *P<0.05, **P<0.01. **(B)** Representative western blot analyses and quantification of the protein levels of genes related to the inflammatory response and p53-mediated apoptosis in HCT116 cells. *P<0.05. **(C)** AAV-shMMP9 or AAV-shNC was injected into *Olfm4*^-/-^ mice or WT littermates one week before DSS treatment. **(D)** Colon sections were stained with H&E (left, scale bar: 50 μm), and inflammation scores (right) were calculated to assess injury and inflammation. TUNEL staining showed apoptotic cells in the colons of mice (scale bar: 50 μm). *P<0.05, **P<0.01, ****P<0.0001. **(E)** The mRNA expression of* Mmp9, Mcp1, Il1β, Icam1, Bcl2,* and *P21* was measured by qRT‒PCR. Data are the means ± SDs. n = 6. *P<0.05, **P<0.01, ***P<0.001, ****P<0.0001. **(F)** The protein levels of IL-1β, IL-6, MCP1, and ICAM1 were measured in serum by ELISAs. Data are the means ± SDs. *n* = 5-8. *P<0.05, **P<0.01, ***P<0.001, ****P<0.0001. **(G)** The protein levels of genes related to the inflammatory response and p53-mediated apoptosis were detected by Western blotting and quantified by ImageJ software. *P<0.05.

Moreover, we found that MMP9 is closely associated with apoptosis-related genes (Figure S6). Deficiency of Mmp9 significantly inhibited the inflammatory response and p53-mediated apoptosis pathway in the LPS-stimulated *Olfm4* knockdown HCT116 and NCM460 cells (Figure 5A and 5B; Figure S7A-S7C). In contrast, overexpression of Mmp9 significantly reversed the suppression of the inflammatory response and p53-mediated apoptosis pathway in the LPS-stimulated *Olfm4*-overexpressing HCT116 cells (Figure S7D-S7G).

To explore whether MMP9 mediated the regulatory effects of OLFM4 on colitis in mice, we transfected *Olfm4*-/- mice and WT littermates with recombinant adeno-associated virus-shMMP9 (AAV-shMMP9) or control vectors (AAV-shNC) through tail vein injection (Figure 5C; Figure S8A-S8D). AAV-mediated inhibition of Mmp9 significantly ameliorated DSS-induced clinical and pathological manifestations of colitis in the *Olfm4*-/- mice (Figure 5D and 5E; Figure S8E and S8F). Inhibition of MMP9 also significantly decreased the inflammatory response, IEC apoptosis, and p53 expression in the DSS-challenged *Olfm4*-/- mice (Figure 5D-5G). These results suggest that MMP9 mediates the regulatory effects of OLFM4 on p53 in colitis.

### OLFM4 targets MMP9 and further regulates p53 via Notch1

**Figure 6:**
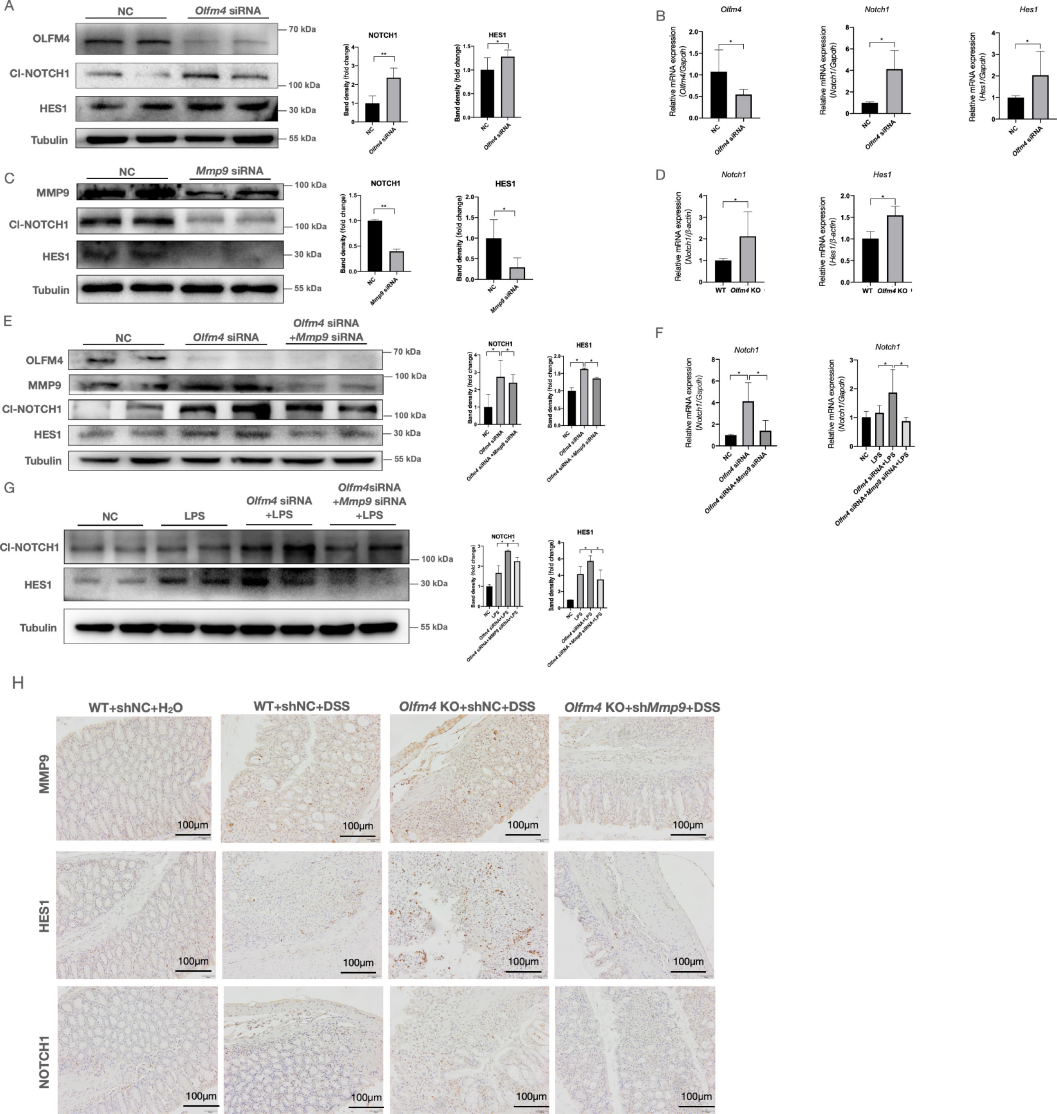
** OLFM4 targets MMP9 and further regulates p53 *via* Notch1. (A)** The protein levels of Cl-NOTCH1 and HES1 increased in OLFM4 knockdown HCT116 cells. *P<0.05, **P<0.01. **(B)** The mRNA expression of *Olfm4, Notch1*, and *Hes1* was measured by qRT‒PCR. Data are the means ± SDs. *n* = 6. *P<0.05. **(C)** The protein levels of Cl-NOTCH1 and HES1 decreased in MMP9 knockdown HCT116 cells. *P<0.05, **P<0.01. **(D)** The mRNA expression of* Notch1* and *Hes1* was detected in mouse colons. Data are the means ± SDs. *n* = 6. *P<0.05. **(E)** The protein levels of OLFM4, MMP9, Cl-NOTCH1, and HES1 detected in HCT116 cells by Western blots and quantified by ImageJ software. *P<0.05. **(F)** The mRNA expression of* Notch1* was measured by qRT‒PCR. Data are the means ± SDs. *n* = 6. *P<0.05. **(G)** The protein levels of Cl-NOTCH1 and HES1 detected in HCT116 cells by Western blots and quantified by ImageJ software. *P<0.05. **(H)** The protein levels of NOTCH1 and HES1 detected by IHC in mouse colons.

As a matrix metalloproteinase, MMP9 functions outside the cell 27. The effects of MMP9 on p53 need to be further clarified. Although MMP9 cannot directly interact with p53 (Figure S9A), previous studies have shown that MMP9 can regulate intestinal epithelial homeostasis through Notch signaling 37, 38, 59. The GSEA scores for Notch signaling positively correlated with MMP9 only in the DSS-treated *Olfm4*-/- mice and not in the WT mice (Figure S9B). Notch signaling could provide support for p53 activation 38, 60. Notch1, together with p53, drives an antiproliferative process of differentiation, senescence, and apoptosis 60. The Notch signaling pathway is a critical regulator of mucosal inflammation and intestinal epithelial cell fate determination 59, 61. Here, we found that knockdown of *Olfm4* significantly increased the mRNA and protein levels of cleaved NOTCH1 and its downstream marker hairy and enhancer of split-1 (HES1) in HCT116 cells (Figure 6A and 6B). Knockdown of Mmp9 significantly suppressed Notch signaling in the *Olfm4* knockdown HCT116 cells regardless of LPS stimulation (Figure 6C-6G). Inhibition of MMP9 also significantly decreased colonic expression of NOTCH1 and HES1 in the DSS-treated *Olfm4*-/- mice (Figure 6H). These findings suggest that OLFM4 targets MMP9 and thereby further regulates the expression of NOTCH1.

**Figure 7:**
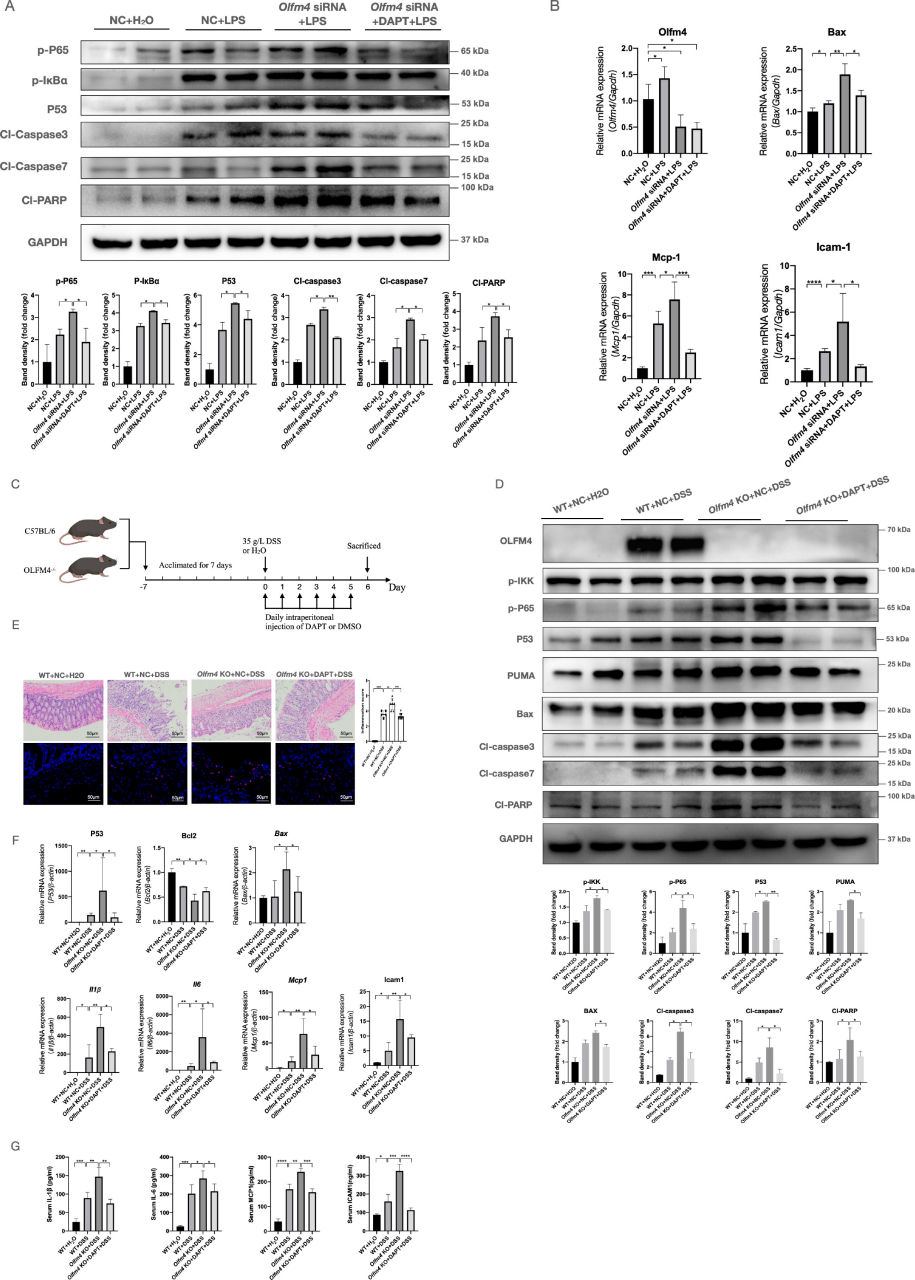
** Inhibiting Notch1 could inhibit the regulatory effect of OLFM4 in colitis. (A)** The protein levels of genes related to the inflammatory response and p53-mediated apoptosis detected in HCT116 cells. *P<0.05, **P<0.01. **(B)** The mRNA expression of *Olfm4, bax, Mcp1,* and *Icam1* was measured by qRT‒PCR. Data are the means ± SDs. n = 6. *P<0.05, **P<0.01, ***P<0.001, ****P<0.0001. **(C)** Mice were injected with DAPT or DMSO every day during the experimental period. **(D)** Representative western blot analyses and quantification of the protein levels of genes related to the inflammatory response and p53-mediated apoptosis detected in distal colons. *P<0.05, **P<0.01. **(E)** Colon sections were stained with H&E (left, scale bar: 50 μm), and inflammation scores (right) were calculated to assess injury and inflammation. TUNEL staining showed apoptotic cells in the mouse colons (scale bar: 50 μm). **P<0.01, ***P<0.001, ****P<0.0001. **(F)** The mRNA expression of* p53, Bcl2, Bax, Il1β, Il6, Mcp1,* and* Icam1* was detected in the distal colons by qRT‒PCR. Data are the means ± SDs. *n* = 6. *P<0.05, **P<0.01. **(G)** The protein levels of IL-1β, IL-6, MCP1, and ICAM1 were measured in serum by ELISAs. Data are the means ± SDs. *n* = 6. *P<0.05, **P<0.01, ***P<0.001, ****P<0.0001.

To further confirm whether NOTCH1 is essential for OLFM4 to regulate colitis, we inhibited the Notch signaling pathway by DAPT47. *In vitro*, 1 μmol/ml DAPT treatment effectively inhibited the Notch signaling pathway (Figure S10A) but did not affect the expression of OLFM4 or MMP9 (Figure S10A) in HCT116 cells. Inhibiting Notch signaling significantly reversed the deteriorating effects of *Olfm4* knockdown on inflammation and p53-mediated apoptosis in LPS-stimulated HCT116 cells (Figure 7A and 7B). *In vivo*, we injected 10 μmol/kg DAPT into WT and *Olfm4*-/- mice every day during the experimental period to inhibit Notch1 (Figure 7C, Figure S10B). We found that DAPT significantly repressed the activity of the p53-mediated apoptosis pathway (Figure 7D-7F) and alleviated DSS-induced colitis in *Olfm4*-/- mice (Figure 7D-7G; Figure S10C-S10E).

### OLFM4 regulates UC in a p53-dependent manner

**Figure 8:**
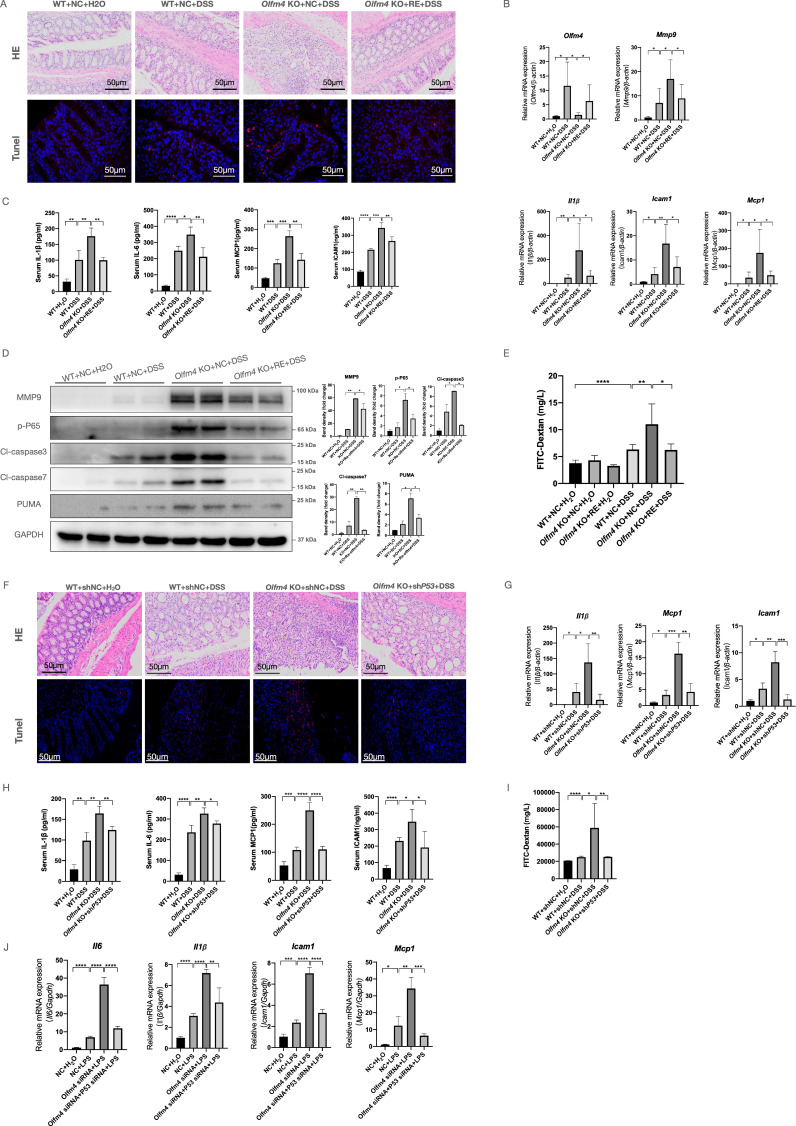
** OLFM4 regulates UC in a p53-dependent manner. (A)** Colon sections were stained with H&E (scale bar: 50 μm) to assess injury and inflammation. TUNEL staining showed apoptotic cells in the mouse colons (scale bar: 50 μm). **(B)** The mRNA expression of *Olfm4*, *Mmp9, Il1β, Icam1,* and* Mcp1* was detected in the distal colons by qRT‒PCR. Data are the means ± SDs. n = 6. *P<0.05, **P<0.01. **(C)** The protein levels of IL-1β, IL-6, MCP1, and ICAM1 were measured in serum by ELISAs. Data are the means ± SDs. *n* = 6. *P<0.05, **P<0.01, ***P<0.001, ****P<0.0001. **(D)** The protein levels of MMP9, p-P65, cl-caspase3, cl-caspase7, and PUMA detected by Western blots in mice. *P<0.05, **P<0.01. **(E)** Serum FITC-dextran levels of DSS-treated WT,* Olfm4^-/-^* mice, and *Olfm4* re-expression mice. Data are the means ± SDs. *n* = 6. *P<0.05, **P<0.01, ****P<0.0001. **(F)** Colon sections were stained with H&E (scale bar: 50 μm) to assess injury and inflammation. TUNEL staining showed apoptotic cells in the mouse colons (scale bar: 50 μm). **(G)** The mRNA expression of *Il1β, Icam1*, and *Mcp1* was detected in the distal colons by qRT‒PCR. Data are the means ± SDs. n = 6. *P<0.05, **P<0.01, ***P<0.001. **(H)** The protein levels of IL-1β, IL-6, MCP1, and ICAM1 were measured in serum by ELISAs. Data are the means ± SDs. *n* = 6. *P<0.05, **P<0.01, ***P<0.001, ****P<0.0001. **(I)** Serum FITC-dextran levels of DSS-treated WT,* Olfm4^-/-^* mice, and p53 knockdown* Olfm4^-/-^* mice. Data are the means ± SDs. *n* = 6. *P<0.05, **P<0.01, ****P<0.0001. **(J)** The mRNA expression of* Il1β, Icam1,* and *Mcp1* was detected in HCT116 cells by qRT‒PCR. Data are the means ± SDs. n = 6. *P<0.05, **P<0.01, ***P<0.001, ****P<0.0001.

To verify whether *Olfm4* is directly involved in the regulation of UC in a p53-dependent manner, we re-expressed *Olfm4* or knocked down p53 expression in *Olfm4*-/- mouse colons by trial vein injection of AAV. We found that the re-expression of *Olfm4* significantly relieved weight loss and colon shortening in the DSS-treated *Olfm4*-/- mice (Figure S11A and S11B). Histological analyses confirmed that re-expression of *Olfm4* significantly ameliorated colitis in the DSS-treated *Olfm4*-/- mice (Figure 8A). Re-expression of *Olfm4* also significantly reversed the upregulation of proinflammatory cytokine and chemokine expression in the colon and serum (Figure 8B and 8C) and increased p-P65 and MMP9 protein levels caused by *Olfm4* deficiency (Figure 8D). In addition, apoptosis and intestinal permeability were improved by *Olfm4* re-expression (Figure 8A, 8D, and 8E). These findings demonstrated that re-expression of *Olfm4* ameliorated DSS-induced colonic inflammation and apoptosis in *Olfm4*-/- mice.

Finally, we found that knocking down p53 significantly ameliorated DSS-induced colitis in *Olfm4*-/- mice (Figure 8F-8H; Figure S11C and S11D). Downregulation of p53 significantly decreased the inflammatory response, IEC apoptosis, and intestinal permeability in the DSS-challenged *Olfm4*-/- mice (Figure 8F and 8I). Consistently, p53 deficiency ameliorated LPS-induced inflammation in Oflm4 knockdown cells *in vitro* (Figure 8J). These results suggest that p53 mediates the regulatory effects of OLFM4 in colitis.

Together, our results suggested that OLFM4 protects against colitis in a p53-dependent manner by targeting MMP9 via Notch1 signaling.

## Discussion

This study focused on the role and regulatory mechanisms of OLFM4 in colitis. We observed a significant increase in *Olfm4* expression in active UC patients and in cellular and mouse models of colitis. We also observed that *Olfm4*-/- mice are more vulnerable to DSS-induced colitis, and OLFM4 regulates colitis through p53-mediated IEC apoptosis. Furthermore, we found that Mmp9 is the key factor connecting *Olfm4* and its downstream pathways through the *Olfm4*-Mmp9-Notch1-p53 axis in colitis.

Previous studies have reported aberrant expression of *Olfm4* in UC patients 21, 23 but have not determined whether the increase is a cause or a consequence of the disease, nor have they investigated the effects and mechanisms of *Olfm4* in UC. We first used more samples than previous studies and detected OLFM4 levels in different segments to confirm that OLFM4 is upregulated in the colonic mucosa of patients with UC. Then, we constructed animal models and cell models to verify the effects of OLFM4 in UC and conducted more in-depth mechanistic studies. The DSS-induced colitis model exhibits several characteristics of human UC, including diarrhea, severe rectal bleeding, weight loss, and infiltration by granulocytes 62. This model has been one of the most extensively used models to study the contribution of innate immune mechanisms in colitis 63. Thus, we chose a DSS-induced acute colitis model to confirm the expression change in *Olfm4*. The expression of OLFM4 was very low in the colon of normal mice, which was consistent with previous studies 11, but it was markedly upregulated in experimental colitis. The induction of *Olfm4* expression coincided with the increased severity of inflammation, as demonstrated by the pathological score evaluated by people who were blinded to the group division. *In vitro*, we observed *Olfm4* upregulation induced by LPS stimulation in the HCT116 cell line, which is commonly used for UC research 42, 64. These data implicate *Olfm4* in the disease progression of colitis.

To better define the role of endogenous *Olfm4* in the development of UC, we established *Olfm4*-/- mice. Interestingly, compared with their WT littermates, *Olfm4*-/- mice showed increased susceptibility to DSS-induced colitis. These data indicate that *Olfm4* plays an inhibitory role in the development of colitis under certain inflammatory stimuli. We also found that *Olfm4* deletion leads to an enhanced immune response and inflammation in the progression of colitis, characterized by enhanced expression of complement Il-1β, Il-6, and Mcp-1 and activation of the NF-κB pathway. NF-κB is an upstream regulator of Mcp1 56, Il-1β, Il-6 65 and C3 66, which can in turn activate NF-κB. Activation of the NF-kB pathway is strongly induced in inflamed tissue from IBD patients 67. In addition, we found that *Olfm4* deficiency promotes IEC apoptosis characterized by enhanced expression of cleaved caspase 3 and cleaved caspase 7 and more TUNEL-positive cells. Beyond apoptosis, other cell death mechanisms, including necroptosis and ferroptosis, also play a key role in the pathogenesis of intestinal injury 68. In contrast to the core apoptotic effectors, the genetic deletion of *Olfm4* did not affect the key necroptotic and ferroptotic machinery, including GPX4, RIPK1, RIPK3 and MLKL 69, 70. OLFM4 is a robust marker for intestinal stem cells 71. In addition to its stem cell properties, it has many roles in different diseases, including anti-inflammation, apoptosis, cell adhesion and proliferation 15, which are more relevant to this study. Previous studies have described the proinflammatory effect of OLFM4 in colon adenocarcinoma, but this study focused on the occurrence of adenocarcinoma, which is very different from ulcerative inflammation 72. In our study, we believe that *Olfm4* is upregulated in the inflammatory state to protect against the development of colitis. We not only confirmed the anti-inflammatory and antiapoptotic roles of OLFM4 in UC but also elucidated its downstream mechanism.

Previous studies have revealed the effect of p53 on epithelial cell apoptosis 33, 36, which was significantly activated in *Olfm4*-/- mice after DSS treatment. The results inspired us to determine whether *Olfm4* can act as an upstream molecule of p53 as an apoptosis regulator in UC. In this study, we found that the p53 protein level was increased in the *Olfm4*-deficient mice and regulated by *Olfm4 in vitro*. We discovered that p53 inhibition could rescue the effect caused by *Olfm4* deficiency. However, there is no direct connection between p53 and *Olfm4*. After searching for potential *Olfm4*-interacting proteins and p53-interacting proteins in the STRING database, we found that Mmp9, an ECM enzyme known to be associated with IBD 27, 28, might be a key factor linking *Olfm4* with p53. Mmp9 is elevated in the intestinal tissue of patients with IBD, and previous studies have reported the role of Mmp9 in the intestinal barrier 73. However, its specific mechanism and its relationship with *Olfm4* have not been studied. Further experiments confirmed our hypothesis that *Olfm4* regulates p53-mediated apoptosis through Mmp9. We first validated the interaction between *Olfm4* and Mmp9. Then, we verified that the interaction between them was required for the function of OLFM4 in IBD.

As the downstream factor of Mmp9, Notch signaling is modulated by Mmp9 and regulates intestinal epithelial homeostasis 59. Previous studies have suggested a potential relationship between *Olfm4* and Notch 74, 75. Our results showed that *Olfm4* can regulate Notch1 rather than just being regulated. Knockdown of p53, reduction in Mmp9, or inhibition of Notch1 could reverse the exacerbation of colitis caused by *Olfm4* deletion. We observed a significant effect of *Olfm4* on the Mmp9-Notch1-p53 axis in colitis regulation. This axis was proposed in research on colitis-associated cancer 37, 38 but has never been assessed in colitis research.

Several limitations are acknowledged in this study. First, OLFM4 may regulate ulcerative colitis through a more complex mechanism than previously discovered. There is crosstalk between p53 and the NF-κB pathway 76. The NF-κB transcription factor can both contribute to and, in other situations, protect against apoptosis 76, 77. The exact role of NF-κB in apoptosis under the specific circumstances of this study needs further investigation. Moreover, other factors may contribute to the pathogenesis in our study. In addition, in our study, we focused more on its effects on pathogenic pathways and less on stem cell properties, which will be an important direction for our future research. Furthermore, because the *Olfm4*-/- mice used in this study were systemically knocked out, it is difficult to avoid the potential influence of OLFM4 in other organs, except the intestine. Therefore, intestinal epithelium-specific OLFM4 knockout mice are planned for future experiments. Finally, further studies are required to demonstrate the clinical application value of *Olfm4* in UC patients.

In conclusion, our results demonstrated that OLFM4 is significantly upregulated in UC, and OLFM4 targets MMP9 and regulates p53-mediated apoptosis via NOTCH1 signaling in experimental colitis. These findings suggest that OLFM4 may serve as a potential diagnostic marker and therapeutic target for UC.

## Supplementary Material

Supplementary figures and tables.

## Abbreviations

AAV: adeno-associated virus
ADP-ribose: apoptosis-related factors including cleaved of poly
ANOVA: analyses of variance
BCL2: B-cell lymphoma 2
BAX: BCL2-associated X protein
CD: Crohn's disease
co-IP: coimmunoprecipitation
DAPT: difluorophenacetyl-L-alanyl-S-phenylglycine t-butyl ester
DMEM: Dulbecco's modified Eagle's medium
DSS: dextran sulfate sodium
ECM: extracellular matrix
ELISA: enzyme-linked immunosorbent assay
FBS: fetal bovine serum
FPKM: fragments per kilobase of transcript per million fragments mapped
FITC: fluorescein isothiocyanate
GSEA: gene set enrichment analysis
HES1: hairy and enhancer of split-1
IBD: inflammatory bowel disease
Icam1: intercellular adhesion molecule-1
IEC: intestinal epithelial cells
Il6: interleukin-6
IHC: immunohistochemistry
IKK: IκB kinase
LPS: lipopolysaccharide
Mcp1: monocyte chemoattractant protein-1
MMP9: matrix metalloproteinase-9
Ngal-MMP9: neutrophil gelatinase B-associated lipocalin and MMP9
NF-κB: nuclear factor-κB
OLFM4: olfactomedin-4
PARP: polymerase
PUMA: p53 upregulated modulator of apoptosis
PBS: phosphate-buffered saline
RPMI: Roswell Park Memorial Institute
SD: standard deviation
SPF: specific pathogen-free
TUNEL: triphosphate nick-end labeling
UC: ulcerative colitis
WT: wild type
