# Supplementary material for: Olfactomedin-4 deletion exacerbates DSS-induced colitis through a matrix metalloproteinase-9-dependent mechanism
Source: Int J Biol Sci. 2023 Apr 9;19(7):2150–66. doi: 10.7150/ijbs.80441 (PMC10158032; doi:10.7150/ijbs.80441)
Supplement: Supplementary file 1 — Supplementary figures and tables. [file ijbsv19p2150s1.pdf]

Supplementary Tables

Table S1 Primer sequences of genes analyzed by Real-time PCR

Gene	Forward primer sequences	Reverse primer sequences
hOLFM4	GAGGGACCAAATCTCCAACT	ATCTGCCACATACAAAGCAT
hIL-1β	AGCTACGAATCTCCGACCAC	CGTTATCCCATGTGTCGAAGAA
hIL-6	ACTCACCTCTTCAGAACGAATTG	CCATCTTTGGAAGGTTCAGGTTG
hMCP-1	CAGCCAGATGCAATCAATGCC	TGGAATCCTGAACCCACTTCT
hICAM-1	GTATGAACTGAGCAATGTGCAAG	GTTCCACCCGTTCTGGAGTC
hMMP9	GGCAGGGACAGTTGCTTCT	CCACGAAACTACCTTCAACTCC
hNOTCH1	GAGGCGTGGCAGACTATGC	CTTGTACTCCGTCAGCGTGA
hHES1	ACACGACACCGGATAAACCAA	GCCGCGAGCTATCTTTCTTCA
hGAPDH	GGAGCGAGATCCCTCCAAAAT	GGCTGTTGTCATACTTCTCATGG
mOLFM4	GTTAGGGTGAAGGAGGAATGA	CTCCTAGACTTCCCTGAAGC
mIL-1β	GCAACTGTTCCTGAACTCAACT	GCAACTGTTCCTGAACTCAACT
mIL-6	GGACCCCAGACAATCGGTTG	GGACCCCAGACAATCGGTTG
mMCP-1	TTAAAAACCTGGATCGGAACCAA	GCATTAGCTTCAGATTTACGGGT
mICAM-1	TGCCTCTGAAGCTCGGATATAC	TCTGTCGAACTCCTCAGTCAC
mMMP9	CTGGACAGCCAGACACTAAAG	CTCGCGGCAAGTCTTCAGAG
mNOTCH1	GATGGCCTCAAAATGGGTACAAG	TCGTTGTTGTTGATGTCACAGT
mHES1	CCAGCCAGTGTCAACACGA	AATGCCGGGAGCTATCTTTCT
mACTB	GGCTGTATTCCCCTCCATCG	CCAGTTGGTAACAATGCCATGT

Table S2 Protein antibodies for Western blot analysis

Protein	Antibody
OLFM4	Cell Signaling Technology, #14369
OLFM4 (mouse specific)	Cell Signaling Technology, #39141
C3	Abcam, #ab200999
P-P65	Santa Cruz, #sc136548
P65	Santa Cruz, #sc8008
P-IκBα	Cell Signaling Technology, #2859
P-IKK	Cell Signaling Technology, #2697
IKKα+β	Abcam, #ab178870
IκBα	Santa Cruz, #1643
P53	Abcam, #ab31333
Cl-NOTCH1	Cell Signaling Technology, # 4147
HES1	Santa Cruz, #166410

Supplementary Figures

Figure S1

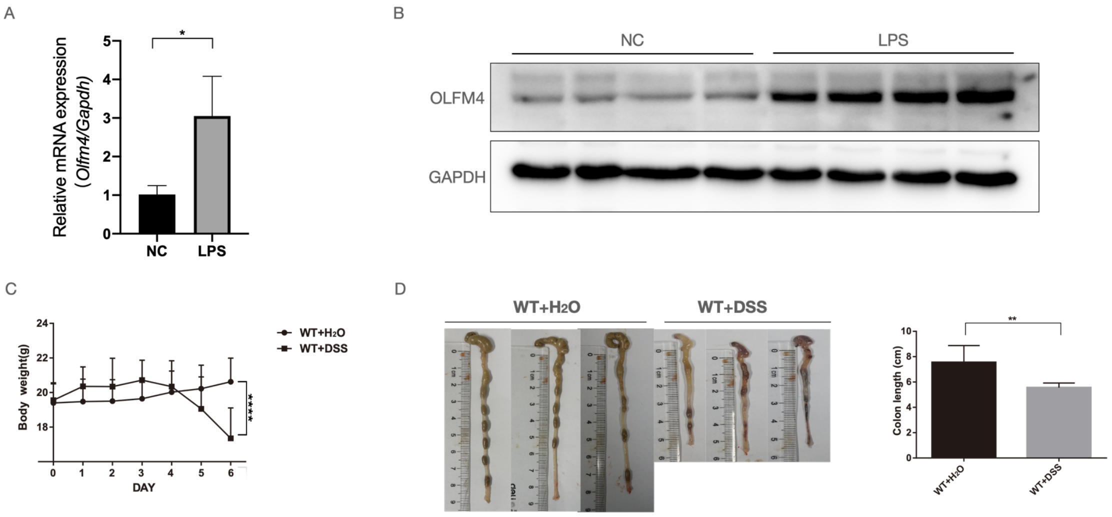

Figure S1. OLFM4 expression in NCM460 cells and DSS induced acute colitis

(A) qRT-PCR analysis of *Olfm4* mRNA expression in LPS-stimulated NCM460 cells. Data are the means ± SD. n = 6. **P*<0.05.

(B) Western blotting analysis of OLFM4 protein level in LPS-stimulated NCM460 cells.

(C) DSS induced significant weight loss. The mice were weighed daily. *****P*<0.0001.

(D) DSS caused a significant reduction in mice colon length. ***P*<0.01.

Figure S2

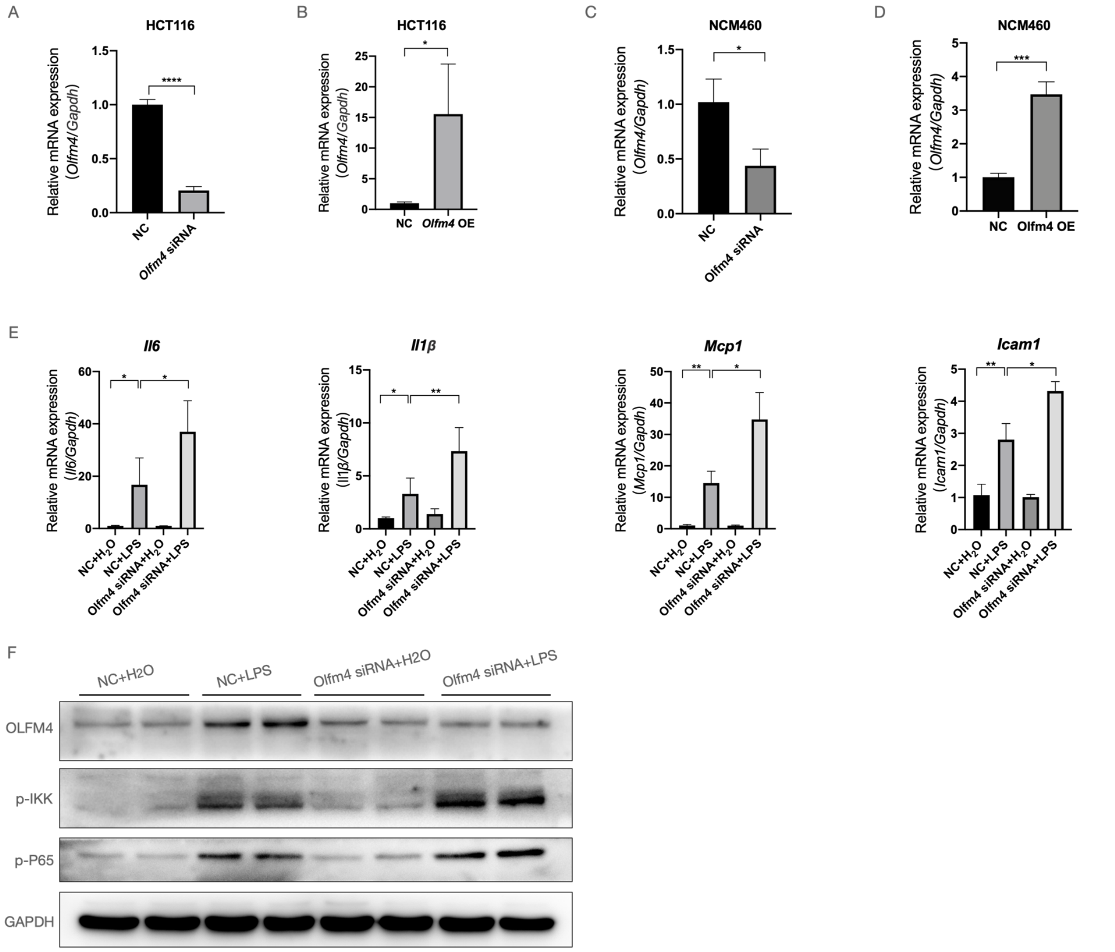

Figure S2. The deletion of Olfm4 exacerbates colitis *in vitro.*

(A) *Olfm4* mRNA expression was knckdowned using *Olfm4* siRNA in the HCT-116 cells. Data are the means ± SD. ****P<0.0001.

(B) *Olfm4* mRNA level was overexpressed using the *Olfm4* plasmid in the HCT-116 cells. Data are the means ± SD. *P<0.05.

(C) *Olfm4* mRNA expression was knckdowned using *Olfm4* siRNA in the NCM460 cells. Data are the means ± SD. *P<0.05.

(D) *Olfm4* mRNA level was overexpressed using the *Olfm4* plasmid in the NCM460 cells. Data are the means ± SD. ***P<0.001.

(E) The mRNA levels of *Il6, Il1β, Mcp1,* and *Icam1* were measured in NCM460 cells by qRT-PCR. Data are the means ± SD. n = 6. *$P<0.05$, **$P<0.01$.

(F) The protein levels of OLFM4, p-P65, p-IKK were measured in NCM460 cells by Western blot

Figure S3

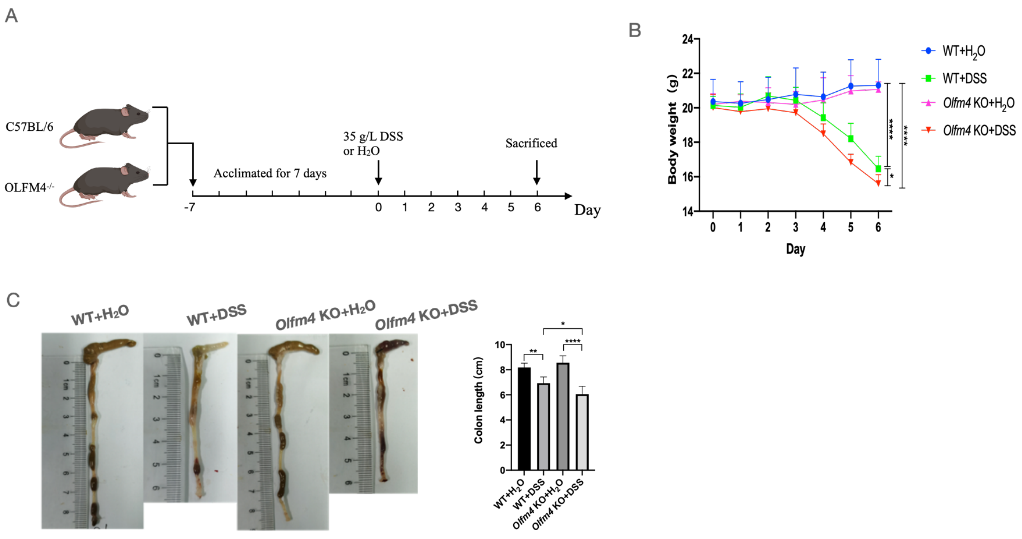

Figure S3. Inflammatory response is exacerbated in OLFM4$^{-/-}$ mice.

(A) WT and *Olfm4*$^{-/-}$ mice were fed dextran sulfate sodium (DSS) in drinking water for 6 days to establish a colitis model.

(B) Mice were weighed daily during the experimental period. **P*<0.05, *****P*<0.0001.

(C) Mice colon length was measured at last day during the experimental. **P*<0.05, ***P*<0.01, *****P*<0.0001.

Figure S4

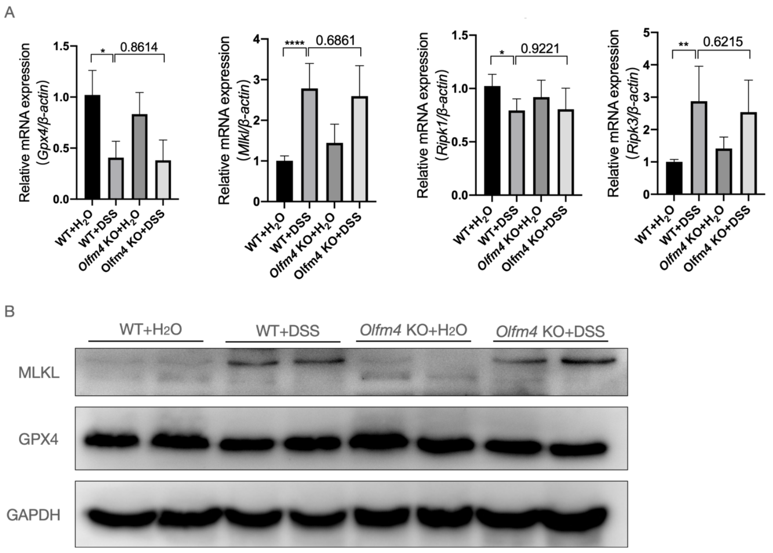

Figure S4. *Olfm4* deficiency did not affect necroptosis and ferroptosis.

(A) The mRNA expression of *Gpx4, Mlkl, Ripk1, and Ripl3* was measured in distal colons by qRT-PCR. Data are the means ± SD. n = 6. **P*<0.05, ***P*<0.01, *****P*<0.0001.

(B) The protein levels of GPX4 and MLKL in distal colons measured by Western blot.

Figure S5

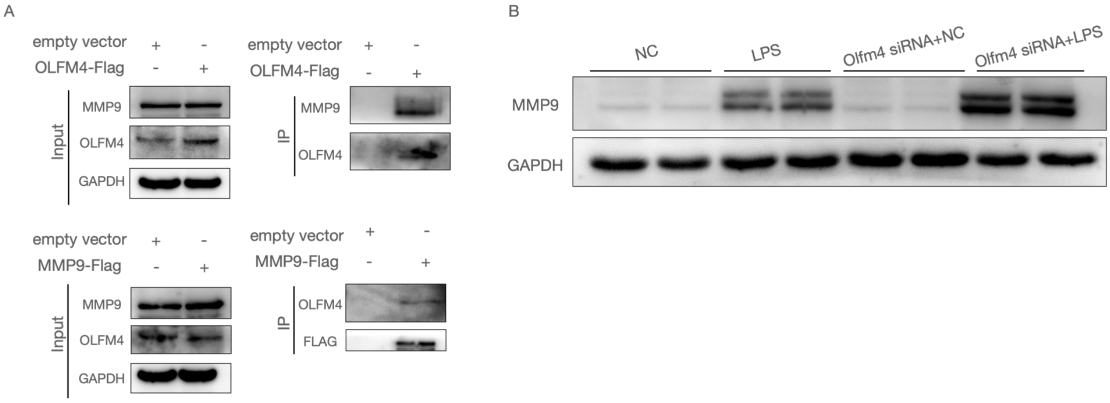

Figure S5. The validation of the interaction between OLFM4 and MMP9

(A) co-IP analysis between OLFM4 and MMP9 in HCT116 cells.

(B) The protein levels of MMP9 in HCT116 cells measured by Western blot.

Figure S6

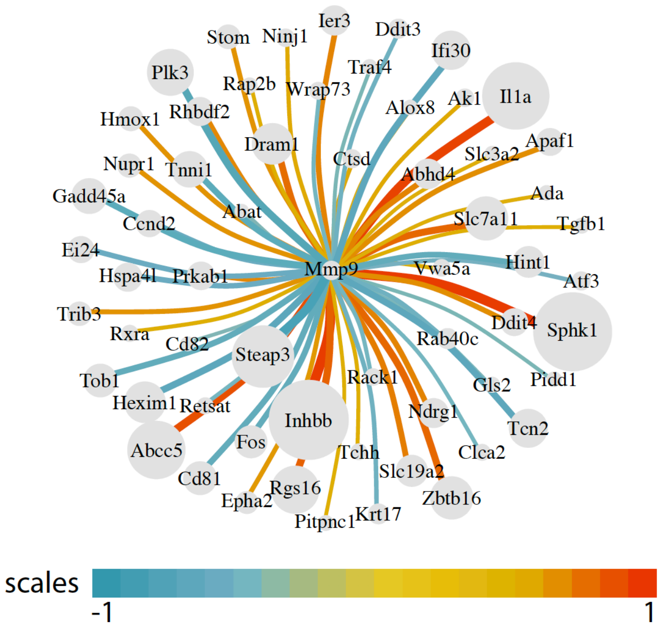

Figure S6. Relationship between MMP9 and apoptosis related genes

Red means positive correlation, while blue means negative correlation

Figure S7

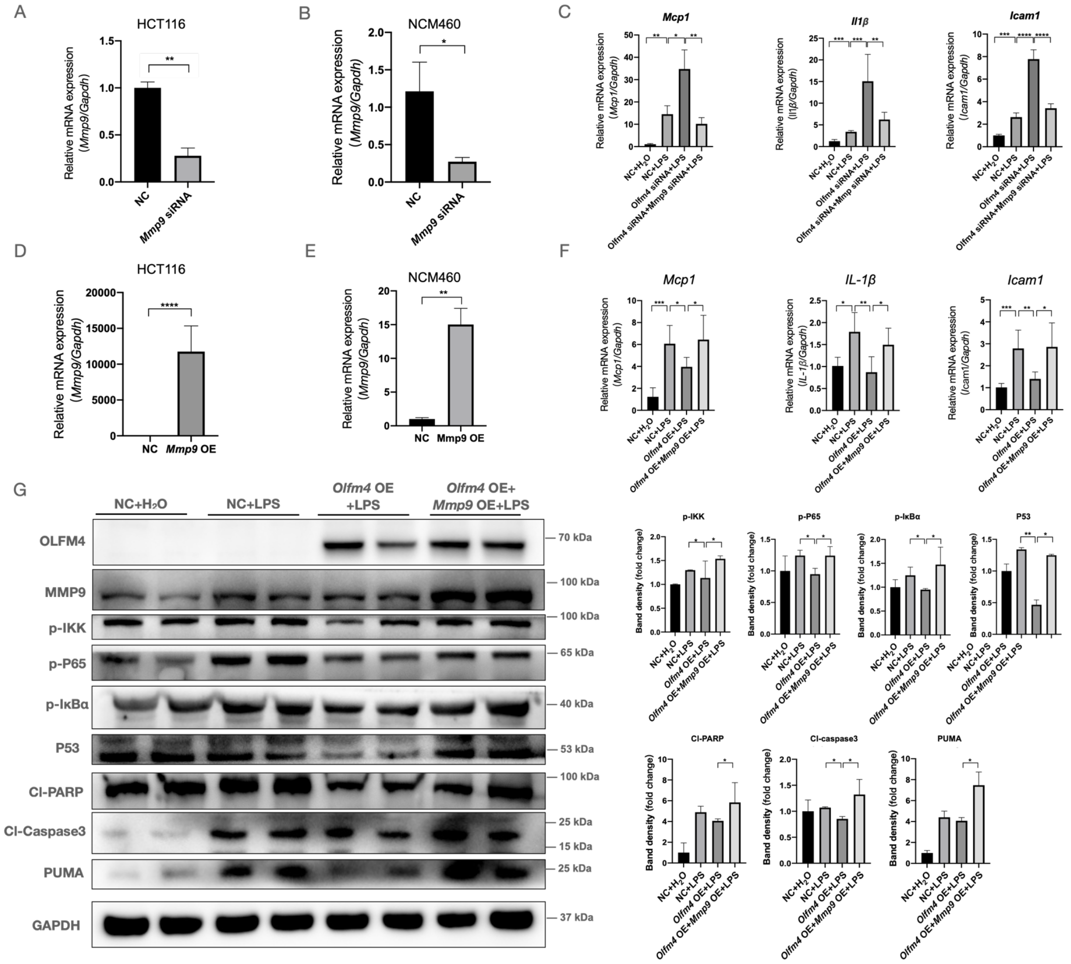

Figure S7. MMP9 mediates the regulatory effect of OLFM4 on p53 *in vitro*

(A) *Mmp9* mRNA expression in the HCT-116 cells assessed by qRT-PCR. Data are the means ± SD. n = 3. **P<0.01.

(B) *Mmp9* mRNA expression in the NCM460 cells assessed by qRT-PCR. Data are the means ± SD. n = 3. *P<0.05.

(C) The mRNA expression of *Mcp1*, *Il1β,* and *Icam1* were detected in the NCM460 cells by qRT-PCR. Data are the means ± SD. n = 6. *P<0.05, **P<0.01, ***P<0.001, ****P<0.0001.

(D) *Mmp9* mRNA expression was overexpressed in the HCT-116 cells assessed by qRT-PCR. Data are the means ± SD. n = 3. ****P<0.0001.

(E) *Mmp9* mRNA expression was overexpressed in the HCT-116 cells assessed by qRT-PCR. Data are the means ± SD. $n = 3$. **P<0.01.

(F) The mRNA expression of *Mcp1*, *Il1β,* and *Icam1* were detected in the HCT-116 cells by qRT-PCR. Data are the means ± SD. $n = 6$. *P<0.05, **P<0.01, ***P<0.001.

(G) Representative western-blot analyses (left) and quantification (right) of the protein levels of genes related to inflammatory response and P53 mediated apoptosis in HCT-116 cells. *P<0.05.

Figure S8

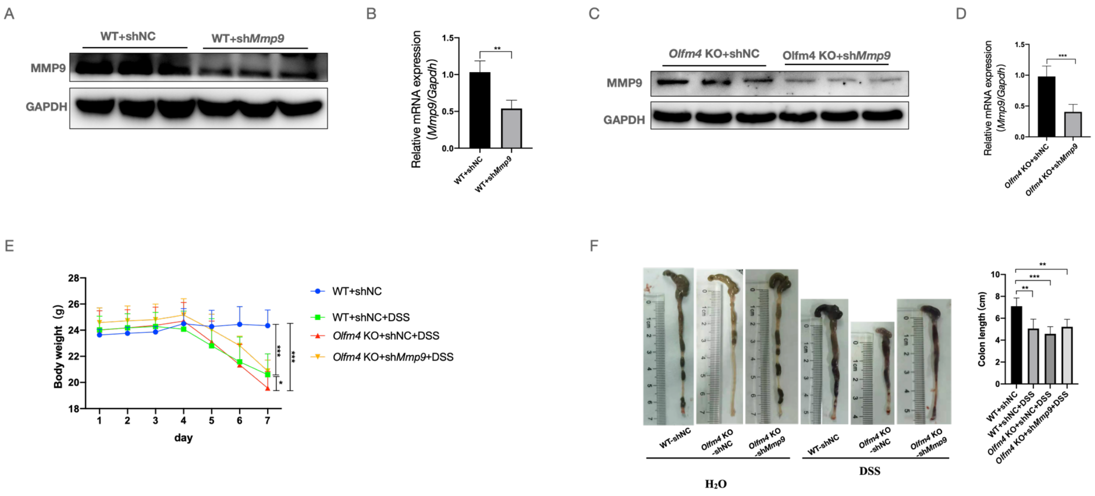

Figure S8. MMP9 mediates the regulatory effect of OLFM4 on p53 in mice colitis.

(A) The protein levels of MMP9 was measured in distal colons of mice by Western blot.

(B) The mRNA expression of *Mmp9* was measured in distal colons by qRT-PCR. Data are the means ± SD. n = 6. $^{**}P<0.01$.

(C) The protein level of MMP9 was measured in distal colons of mice by Western blot.

(D) The mRNA expression of *Mmp9* was measured in distal colons by qRT-PCR. Data are the means ± SD. n = 6. $^{***}P<0.001$.

(E) Mice were weighed daily during the experimental period. $^{*}P<0.05$, $^{***}P<0.001$.

(F) Mice colon length was measured at last day during the experimental. $^{**}P<0.01$, $^{***}P<0.001$.

Figure S9

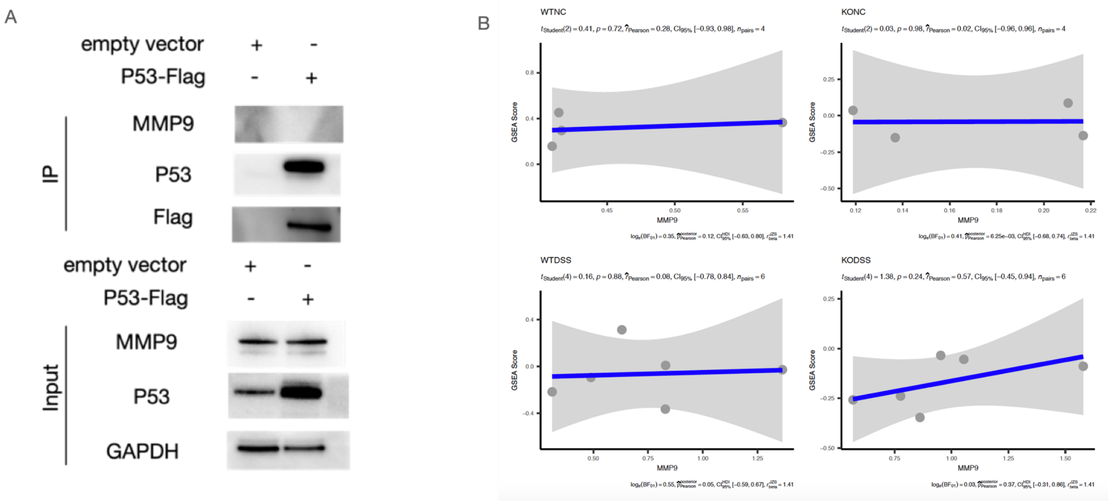

Figure S9. Notch1 may mediated the association between MMP9 and p53

(A) MMP9 cannot directly interact to p53.

(B) The relationship between GSEA scores for Notch signaling and MMP9.

Figure S10

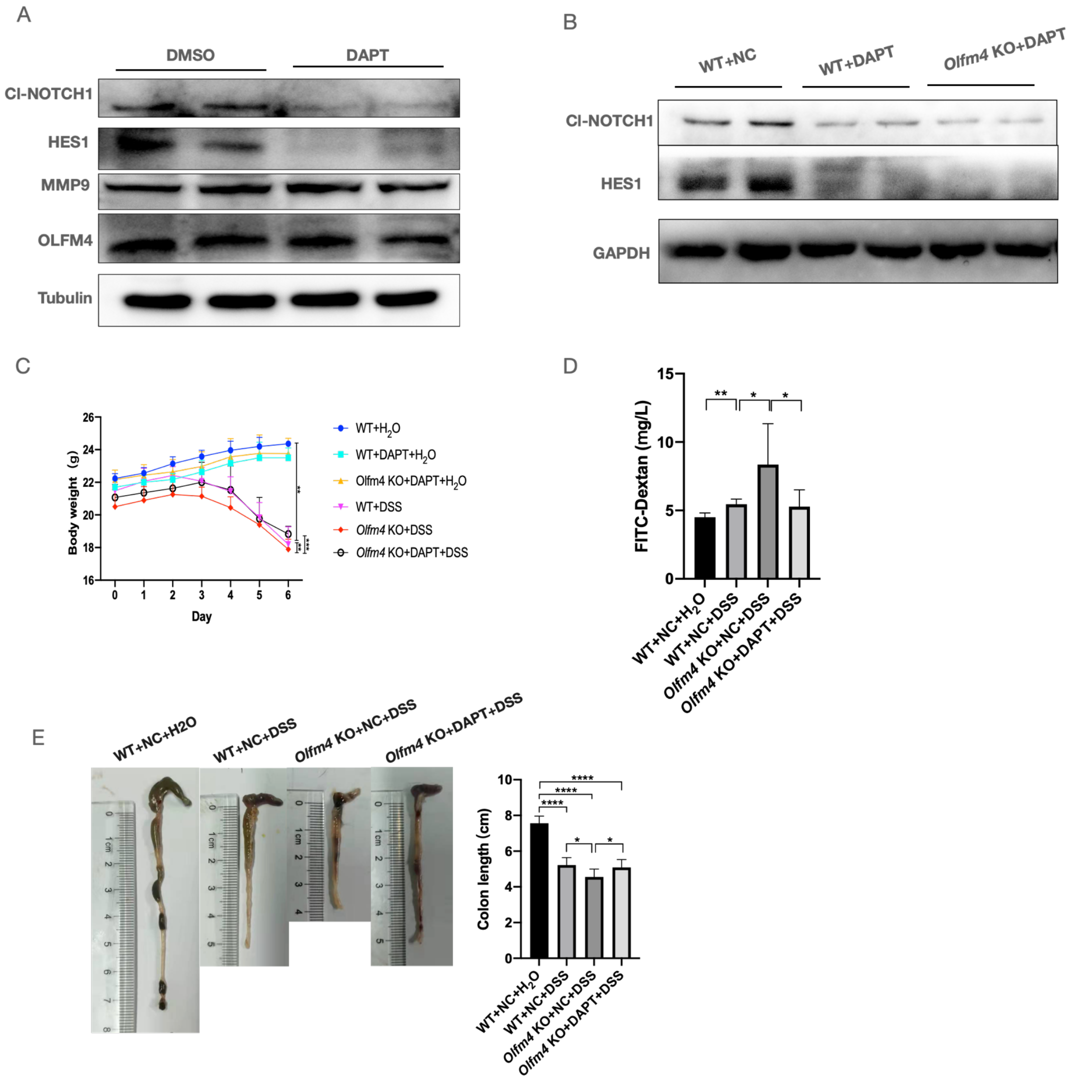

Figure S10. Inhibiting Notch1 could inhibit the regulate effect of OLFM4

(A) The protein levels of Cl-NOTCH1, HES1, MMP9 and OLFM4 in DAPT treated HCT-116 cells.

(B) The protein levels of Cl-NOTCH1 and HES1 were decreased by DAPT in WT mice colons.

(C) Mice were weighed daily during the experimental period.

(D) Serum FITC–dextran levels of mice gavaged with FITC-dextran 4 h before sacrifice. *P<0.05, **P<0.01.

(E) Mice colon length was measured at last day during the experimental. n = 6, *P<0.05, ****P<0.0001

Figure S11

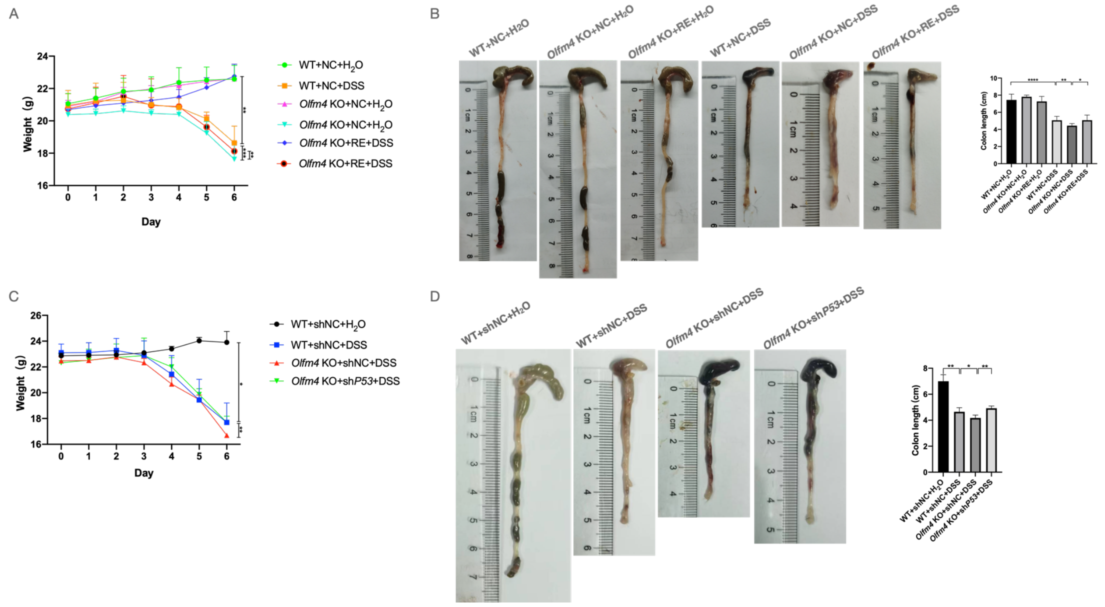

Figure S11. Weight and colon length of mice

(A) Mice were weighed daily during the experimental period. ***P*<0.01, ****P*<0.001.

(B) Mice colon length was measured at last day during the experimental. $n = 6$, **P*<0.05, ***P*<0.01, *****P*<0.0001.

(C) Mice were weighed daily during the experimental period. **P*<0.05, ***P*<0.01.

(D) Mice colon length was measured at last day during the experimental. $n = 6$, **P*<0.05, ***P*<0.01.
